# African Swine Fever Virus Infection and Cytokine Response In Vivo: An Update

**DOI:** 10.3390/v15010233

**Published:** 2023-01-14

**Authors:** Giulia Franzoni, Miriam Pedrera, Pedro J. Sánchez-Cordón

**Affiliations:** 1 Department of Animal Health, Istituto Zooprofilattico Sperimentale della Sardegna, 07100 Sassari, Italy; 2 Centro de Investigación en Sanidad Animal (CISA), Instituto Nacional de Investigación y Tecnología Agraria y Alimentaria (INIA), Consejo Superior de Investigaciones Científicas (CSIC), Valdeolmos, 28130 Madrid, Spain

**Keywords:** African swine fever, cytokines, interleukins, interferons, chemokines

## Abstract

African swine fever (ASF) is a hemorrhagic viral disease of domestic pigs and wild suids (all *Sus scrofa*) caused by the ASF virus (ASFV). The disease is spreading worldwide without control, threatening pig production due to the absence of licensed vaccine or commercially available treatments. A thorough understanding of the immunopathogenic mechanisms behind ASFV infection is required to better fight the disease. Cytokines are small, non-structural proteins, which play a crucial role in many aspects of the immune responses to viruses, including ASFV. Infection with virulent ASFV isolates often results in exacerbated immune responses, with increased levels of serum pro-inflammatory interleukins (IL-1α, IL-1β, IL-6), TNF and chemokines (CCL2, CCL5, CXCL10). Increased levels of IL-1, IL-6 and TNF are often detected in several tissues during acute ASFV infections and associated with lymphoid depletion, hemorrhages and oedemas. IL-1Ra is frequently released during ASFV infection to block further IL-1 activity, with its implication in ASFV immunopathology having been suggested. Increased levels of IFN-α and of the anti-inflammatory IL-10 seem to be negatively correlated with animal survival, whereas some correlation between virus-specific IFN-γ-producing cells and protection has been suggested in different studies where different vaccine candidates were tested, although future works should elucidate whether IFN-γ release by specific cell types is related to protection or disease development.

## 1. Introduction

African swine fever (ASF) is a viral hemorrhagic disease of domestic pigs and wild suids (all *Sus scrofa*), caused by ASF virus (ASFV), a large, enveloped double-stranded DNA virus belonging to the *Asfarviridae* family [1]. Since cases of ASF caused by isolates originating from Southeast Africa and belonging to genotype II were described in Georgia in 2007, the disease has spread without control affecting different countries of Eastern and Western Europe, and also, since 2018, the world’s largest pig producer, China. The disease has also reached Oceania, with cases described in Papua New Guinea in 2020, and most recently the Americas, with outbreaks affecting the Dominican Republic and Haiti since 2021 [2]. Without doubt, ASF represents the biggest threat the swine industry has faced in decades [3]. The available control measures have failed to eliminate ASF in most affected countries. Currently, there are no safe and effective licensed vaccines or other treatment options, thus ASF control relies solely on veterinary control measures, such as culling of affected animals and establishment of restriction zones, as well as strong passive and active surveillance [4]. There is an urgent need to create safe and efficient countermeasures to avoid the economic losses associated with ASF outbreaks. In this sense, a thorough understanding of the immunopathogenic mechanisms behind ASFV infection is essential to develop new tools focused on preventing and controlling the disease.

Cytokines are small, non-structural proteins, which affect almost all biological processes and play a crucial role in many aspects of the immune response. They influence non-specific responses to infection and specific responses to antigens, orchestrating both the innate and adaptive immune response [5]. The term cytokines encompasses interleukins (ILs), interferons (IFNs), chemokine family and tumor necrosis factor (TNF) family, as well as various growth factors [5,6]. In pigs, 42 genes have been classified as IL, 21 as CCL chemokines and 16 as CXCL chemokines. Additionally, 12 TNF superfamily proteins have been identified in this species, along with 30 transforming growth factor (TGF)-β superfamily members, whereas porcine type I IFNs comprise at least 39 functional genes [6]. Most of the time, cytokines act as protectors against both intrinsic and extrinsic noxious stimuli, such as invading pathogens. Nevertheless, an exaggerated immune activation accompanied by an excessive cytokine release, known as a “cytokine storm”, has been associated with several diseases, including viral hemorrhagic diseases. Cytokine production should subside as the infection is eliminated, otherwise these soluble factors would drive the host into a state of chronically activated cells with dangerous consequences [5].

Over the last years, cytokines have become important diagnostic, prognostic and therapeutic agents in human and animal diseases [5,6]. This review aims to summarize the current knowledge on ASFV infection and cytokine response in vivo. In the first section of the review, current knowledge about the concentrations and evolution of circulating pro-inflammatory and anti-inflammatory ILs, IFNs, key chemokines and growth factors will be approached. Next, the secretion and expression of key cytokines in different tissues during infections with either attenuated or virulent isolates will be also addressed. We aim at providing a clear portrait of how ASFV modulates these soluble factors, facilitating a more comprehensive understanding of pathogen–host interactions, hopefully contributing the current effort to design safe and efficient vaccines or treatments against this fatal hemorrhagic viral disease.

## 2. Circulating Levels of Cytokines during ASFV Infection In Vivo

In the first section of this review, we summarize the available knowledge in the literature on circulating levels of different cytokines during ASFV infection.

### 2.1. Interleukin (IL)-1 Superfamily

In pigs, eleven IL-1 superfamily members have been identified: IL-1α, IL-1β, IL-1β2, IL-18, IL-1Ra, IL-33, IL-36α, IL-36β, IL-36γ, IL-36Ra, IL-1F10 (IL-38) [6]. In humans, the IL-1 superfamily also includes IL-37, which is present in several veterinary species including cows, sheep, dogs and horses, although it is a pseudogene in pigs [6]. IL-1F10, IL-1Ra, IL-36Ra are receptor antagonists, whereas IL-1α, IL-1β, IL-18, IL-33, IL-36α, IL-36β, IL-36γ are receptor agonists [7]. IL-1 superfamily members are secreted by innate immune cells upon infection or tissue damage, regulating many aspects of both innate and adaptive immune responses [8].

Changes in the levels of four of these cytokines during ASFV infection (IL-1α, IL-1β, IL-18, IL-1Ra) were monitored in previous studies. The main results of these studies will be described below (see summary in Table 1).

#### 2.1.1. IL-1α and IL-1β

IL-1α and IL-1β are pro-inflammatory cytokines with similar biological properties, produced and released at the early stages of the immune response to infections. These cytokines are also pathogenic mediators of autoinflammatory, autoimmune, infectious and degenerative diseases [7].

IL-1α enhances the production of several chemokines, promoting infiltration of neutrophils and monocytes in the inflamed tissue [9]. This cytokine is constitutively expressed in several cell types and is released into the extracellular space in case of cell death. It can also be synthesized de novo and can be actively secreted or passively released from apoptotic cells [10]. Several studies have suggested that IL-1α production is under the control of the related cytokine, IL-1β [9].

IL-1β is a potent pro-inflammatory cytokine, which is produced and released at the early stages of infections, stressful situations or lesions. Monocytes and macrophages are the main source of this cytokine, but it is also released by natural killer (NK) cells, B cells, dendritic cells (DCs), fibroblasts and epithelial cells [10]. IL-1β is a powerful endogenous pyrogen. During inflammation, IL-1β triggers the production of acute phase proteins (APP) in the liver and acts on the central nervous system to induce fever and prostaglandin secretion [9,10]. It is also a chemoattractant for neutrophils and enhances the expansion and differentiation of different CD4^+^ T cell subsets (Th1, Th2 and Th17) [11]. IL-1β promotes angiogenesis, increasing the expression of cell adhesion molecules on leukocytes and endothelial cells, and also activates infiltrated cells to produce endothelial cell activating factors such as VEGF. IL-1β is synthesized as a leaderless precursor, or from an inactive IL-1β precursor that must be cleaved by inflammasome-activated caspase-1. After activation, autophagy plays a major role in the release of this cytokine [10].

Infections with virulent ASFV isolates often results in increased levels of serum pro-inflammatory cytokines such as IL-1α and IL-1β [12]. Increased levels of IL-1β were first described in domestic pigs infected with a high dose of the virulent genotype I E70 [13,14]. Subsequent studies have also reported an increase in circulating levels of these two pyrogenic cytokines in domestic pigs infected with virulent isolates belonging to either genotype I (Benin97/1) [15] or genotype II (Armenia07) [16], Armenia08 [17], SY18 [12] or HLJ/18 [18]. No changes in IL-1β circulating levels were instead observed in pigs immunized with moderately virulent isolates, such as Netherland’86 [19], or attenuated isolates, such as BeninΔMGF, OURT88/3, Pret4∆9GL, ASFV-G-Δ9GLΔUK and HLJ/18-7GD [15,18,20,21,22]. Surprisingly, immunizations with the attenuated mutant ASFV-Δ7R, lacking MGF505-7R, but not immunizations with its wild type strain (HJL/18), resulted in increased serum concentrations of IL-1β [23]. Differences with the other attenuated ASFV deletion mutants might be linked to peculiar function of the MGF gene.

Instead, only a few studies have analyzed the modulation of the related cytokine IL-1α. Circulating levels of this cytokine seem to mirror those of IL-1β. In detail, researchers observed that circulating levels of this cytokine rose in pigs infected with the highly virulent isolates, Armenia08 [17] and HLJ/18 [18], but not when pigs were infected with the attenuated deletion mutants, Pret4∆9GL, ASFV-G-Δ9GLΔUK or HLJ/18-7GD [18,21,22]. Surprisingly, there were no increases in the levels of either IL-1α or IL-1β after infection with the virulent isolate SY18 or its attenuated derived mutant, SY18ΔL7-11. However, in a study, animals infected with SY18 presented a sustained increase of IL-1Ra circulating levels [24].

Future studies should also explore whether the mass release of IL-1Ra might be responsible for the lower release of the related pro-inflammatory cytokines IL-1α and IL-1β. A recent study has reported interesting differences in serum values of both cytokines in pigs with different immunological and hygienic status. In this sense, farm pigs released higher levels of several pro-inflammatory cytokines, including IL-1α and IL-1β, compared to specific-pathogen-free (SPF) pigs in response to the moderately virulent isolate Estonia2014 (genotype II). Such an increase was accompanied by lower serum values of anti-inflammatory IL-1Ra (described later in Section 2.1.3) and increased mortality [17].

In most of the studies mentioned above, researchers observed that values of IL-1α and IL-1β started to rise at early stages after infection with virulent ASFV isolates, and that serum concentrations often peaked at the end of the observation period [12,13,14,16,17]. The release of these cytokines in vivo should enhance immune surveillance, promoting viral clearance and the development of the acquired immune response [10]. However, steady high serum levels of these proinflammatory cytokines for long periods of time after virus infection were also suggested as indicative of exaggerated, aberrant and failed immune activation with fatal consequences for the outcome of the disease, and with a principal role in the pathogenic mechanisms of ASF lesions [25,26].

#### 2.1.2. IL-18

IL-18 is another member of the IL-1 superfamily. It is mainly produced by macrophages, in particular classically activated macrophages, as well as DCs and epithelial cells, such as keratinocytes [8]. Pro-IL-18 is constitutively expressed, although it must be processed by caspase 1 in order to be activated. This cytokine binds to the receptor IL-18R, which leads to the recruitment of IL-18RAP, which initiates intracellular signaling cascade [8]. IL-18 is potent inducer of IFN-γ production. It synergizes with IL-12 to activate cytotoxic T cells and NK cells [10] and plays a crucial role in the T helper cell type 1 (Th1) response during immune recognition [27,28]. In addition, IL-18 can enhance other T-cell responses, such as Th17, in synergy with IL-23, or Th2 responses in the absence of IL-12, IL-15 or IL-23 [29]. Finally, it has been reported that this cytokine is not pyrogenic, and that it can also attenuate the febrile response induced by the pyrogenic IL-1β [28].

Despite the crucial role of this cytokine in the development of the protective cell-mediated immune response, only some studies have investigated its modulation during ASFV infections. In two different studies carried out in domestic pigs infected with the genotype II highly virulent isolates SY18 [12,24] and Armenia08 [17], results revealed a trend of sustained increase of IL-18 serum values, which was concomitant with the increase of other pro-inflammatory cytokines, although such an increase was not detected in all evaluated animals [24]. Immunizations with the attenuated isolate SY18ΔL7-11 or the moderately virulent isolate Estonia2014 triggered IL-18 release in a similar manner, although with substantial differences between analyzed pigs [17,24].

#### 2.1.3. IL-1Ra

The IL-1 system must be tightly controlled to prevent potentially pathological over-response to stressors. It is indeed regulated at several levels, including receptor antagonists [7]. IL-1Ra binds the receptor IL-1R1 with affinity higher than that of IL-1α or IL-1β, but it does not trigger the association of IL-1 receptor accessory protein, thus there is no activation of the IL-1 signaling [7]. Overall, IL-1Ra is released to block further IL-1 activity, preventing the development of inflammation and of an exacerbated immune response [10].

To date, only a few studies have evaluated ASFV’s impact on circulating levels of this cytokine. In one of these studies, results showed that infection of domestic pigs with the genotype II virulent isolate SY18 (10^3^ TCID_50_/animal inoculated by intramuscular-IM route) resulted in increased levels of this cytokine, reaching the highest values on the day before death. In the same study, the levels of this anti-inflammatory cytokine were also monitored in pigs infected with the attenuated isolate SY18ΔL7-11 (10^3^ or 10^6^ TCID_50_/animal; IM route). Results revealed that levels of IL-1Ra increased soon after infection, but returned to baseline levels at the time of challenge at day 28 post-infection (pi) [24]. Recent studies have reported that serum values of this cytokine increased soon after infection with both highly the virulent isolate Armenia08 and moderately virulent isolate Estonia2014 (infected with 3–6 × 10^2^ TCID_50_/animal by IM route) [17]. In such study, interesting differences were also observed between pigs with different immunological and hygienic status in response to Estonia2014 immunization.

**Table 1:** Modulation of serum levels of IL-1α, IL-1β, IL-18, IL-1Ra in domestic pigs infected with ASFV isolates of diverse virulence.

IL-1 Superfamily Member	ASFV Isolate *	Isolate Virulence	Dose/Route of Inoculation	Day Post-Inoculation (dpi) Analyzed	Impact on Cytokine’s Serum Values	Reference
**IL-1α**	Pret4∆9GL *	Attenuated	10^4^ TCID_50_, IM	7, 10, 14	None	[21]
	ASFV-G-Δ9GLΔUK *	Attenuated	10^4^ TCID_50_, IM	7, 14	None	[22]
	SY18	Highly virulent	10^3^ TCID_50_, IM	0, 3, 7, 10, 14, 21	None	[24]
	SY18ΔL7-11 *	Attenuated	10^3^ or 10^6^ TCID_50_, IM	0, 3, 7, 10, 14, 21, 28	None	[24]
	Armenia08	Highly virulent	0.6–1.2 × 10^2^ TCID_50_, IM	1, 2, 3, 4, 5, 6, 7	Raise	[17]
	Estonia2014	Moderately virulent	0.6–1.2 × 10^2^ TCID_50_, IM	2, 4, 5, 7, 9, 11, 14	Raise mainly in farm pigs	[17]
	HLJ/18	Highly virulent	10^6^ TCID_50_, IM	7	Raise	[18]
	HLJ/18-7GD *	Attenuated	10^6^ TCID_50_, IM	0, 4, 7, 14, 20, 28	None	[18]
**IL-1β**	España-70	Highly virulent	10^5^ TCID_50_, IM	2, 4, 6	Raise	[13]
	España-70	Highly virulent	10^5^ TCID_50_, IM	2, 4, 6	Raise	[14]
	Armenia07	Highly virulent	10^4^ HAD_50_	0, 1, 2, 3, 4, 5, 6, 7	Raise	[16]
	Pret4∆9GL *	Attenuated	10^4^ TCID_50_, IM	7, 10, 14	None	[21]
	ASFV-G-Δ9GLΔU *	Attenuated	10^4^ TCID_50_, IM	7, 14	None	[22]
	Netherland’86	Moderately virulent	2 × 10^3.5^ or 2 × 10^5.5^ TCID_50_, IN	0, 7, 10, 17, 27	None	[19]
	Benin∆MFG *	Attenuated	10^3^ TCID_50_, IM (boost at 21 dpi with same dose/route)	0, 2, 4, 7, 10, 15, 21, 24, 28, 39	Raise only in one pig	[15]
	Benin∆MFG *	Attenuated	10^3^ TCID_50_, IN (boost at 21 dpi with same dose/route)	0, 2, 4, 7, 10, 15, 21, 24, 28, 39	None	[15]
	Benin97/1	Highly virulent	10^4^ TCID_50_, IM	0, 3, 5	Mild raise	[15]
	SY18	Highly virulent	10^3^ TCID_50_, IM	0, 1, 2, 3, 4, 5, 6, 7, 8	Raise	[12]
	SY18	Highly virulent	10^3^ TCID_50_, IM	0, 3, 7, 10, 14, 21	None	[24]
	SY18ΔL7-11 *	Attenuated	10^3^ or 10^6^ TCID_50_, IM	0, 3, 7, 10, 14, 21, 28	None	[24]
	HLJ/18	Highly virulent	10^3^ HAD_50_, IM	1, 5, 8	None	[23]
	ASFV-Δ7R *	Attenuated	10^3^ or 10^5^ TCID_50_, IM	1, 5, 8	Raise	[23]
	Armenia08	Highly virulent	0.6–1.2 × 10^2^ TCID_50_, IM	1, 2, 3, 4, 5, 6, 7	Raise	[17]
	Estonia2014	Moderately virulent	0.6–1.2 × 10^2^ TCID_50_, IM	2, 4, 5, 7, 9, 11, 14	Raise mainly in farm pigs	[17]
	HLJ/18	Highly virulent	10^6^ TCID_50_, IM	7	Raise	[18]
	HLJ/18-7GD *	Attenuated	10^6^ TCID_50_, IM	4, 7, 14, 20, 28	None	[18]
**IL-18**	Netherland’86	Moderately virulent	2 × 10^3.5^ or 2 × 10^5.5^ TCID_50_, IN	0, 7, 10, 17, 27	None	[19]
	SY18	Highly virulent	10^3^ TCID_50_, IM	0, 1, 2, 3, 4, 5, 6, 7, 8	Raise	[12]
	SY18	Highly virulent	10^3^ TCID_50_, IM	0, 3, 7, 10, 14, 21	None	[24]
	SY18ΔL7-11 *	Attenuated	10^3^ or 10^6^ TCID_50_, IM	0, 3, 7, 10, 14, 21, 28	None	[24]
	Armenia08	Highly virulent	0.6–1.2 × 10^2^ TCID_50_, IM	1, 2, 3, 4, 5, 6, 7	Raise	[17]
	Estonia2014	Moderately virulent	0.6–1.2 × 10^2^ TCID_50_, IM	2, 4, 5, 7, 9, 11, 14	Raise mainly in farm pigs	[17]
**IL-1Ra**	SY18	Highly virulent	10^3^ TCID_50_, IM	0, 3, 7, 10, 14, 21	Raise	[24]
	SY18ΔL7-11 *	Attenuated	10^3^ or 10^6^ TCID_50_, IM	0, 3, 7, 10, 14, 21, 28	Mild raise	[24]
	Armenia08	Highly virulent	0.6–1.2 × 10^2^ TCID_50_, IM	1, 2, 3, 4, 5, 6, 7	Raise	[17]
	Estonia2014	Moderately virulent	0.6–1.2 × 10^2^ TCID_50_, IM	2, 4, 5, 7, 9, 11, 14	Raise mainly in SPF pigs	[17]
	HLJ/18	Highly virulent	10^6^ TCID_50_, IM	7	Raise	[18]
	HLJ/18-7GD *	Attenuated	10^6^ TCID_50_, IM	4, 7, 14, 20, 28	None	[18]

* Deletion mutant; IM: intramuscular inoculation; IN: intranasal inoculation; TCID: tissue culture infectious dose; HAD: hemadsorption dose; SPF: specific pathogen free.

SPF pigs released higher levels of IL-1Ra at earlier stages post-infection (day 4 pi) than farm pigs. Such an increase in IL-1Ra levels was concomitant with lower serum values of pro-inflammatory cytokines and reduced mortality [17]. In another recent study, increased serum levels of this cytokine were described in pigs infected with the virulent isolate HLJ/18, but not in pigs infected with its derived attenuated mutant HLJ/18-7GD [18]. These studies suggested that IL-1Ra would play an important role in ASFV immunopathology, thus future studies should include this receptor antagonist in the panel of circulating cytokines that should be monitored during ASFV experimental studies.

### 2.2. Interleukin (IL)-6

IL-6 is a member of the IL-6 superfamily, which in pigs also includes cardiotrophin (CTF)1, CTF2, colony stimulator factor (CSF)3, IL-11, IL-23A and IL-31 [6]. It is a pleiotropic cytokine that exerts both pro-inflammatory and anti-inflammatory properties [10]. IL-6 plays a crucial role in several processes, spanning from immunity to regulation of metabolic, regenerative and neuronal processes. IL-6 is also an endogenous pyrogen, which promotes fever and the production of APP in the liver [10,30]. Proinflammatory properties, including recruitment of monocytes to the inflammation site and maintenance of Th17 cells, are elicited when IL-6 signals through the trans pathway via soluble IL-6 receptors (sIL-6R) binding to transmembrane transducer protein gp130. On the contrary, regenerative or anti-inflammatory properties, such as inhibition of liver inflammation and insulin resistance, are elicited when IL-6 signals through the classical pathway, which occurs via the IL-6 receptor (IL-6R) [10,30].

So far, only a few research works have studied ASFV’s impact on IL-6 circulating levels. Some studies described increased levels of IL-6 after infection with virulent genotype II ASFV isolates, alongside other pro-inflammatory cytokines, with peaks at the end of the observation period at day 7 pi [12,16,17,18]. Other studies described no modulation of serum levels of this cytokine in pigs inoculated with moderately virulent isolates (Netherland’86) or attenuated strains (SY18ΔL7-11, HLJ/18-7GD) [18,19,24]. These studies suggested that increased serum levels of IL-6 were a hallmark of a sustained inflammatory response occurring in acute ASFV infections induced by highly virulent isolates, which was absent in animals immunized with attenuated strains. Surprisingly, in one study in which pigs were infected with the highly virulent isolate SY18 (genotype II), an increase of circulating levels of IL-6 was not observed [24].

### 2.3. Tumor Necrosis Factor (TNF)

Tumor necrosis factor (TNF; formerly known as TNF-α) is one of the twelve members of the TNF superfamily (TNFSF), which also includes TNFSF9, TNFSF10, TNFSF11, TNFSF12, TNFSF13, TNFSF13B, TNFSF14, TNFSF15, lymphotoxin alpha (LTA), lymphotoxin beta (LTB) and ectodysplasin A (EDA) [6].

TNF is a pro-inflammatory cytokine mainly produced by macrophages [31]. This cytokine is a potent pyrogenic, usually released early in response to infection, able to induce fever, cachexia and inflammation. TNF triggers the synthesis of APP in the liver, such as C reactive protein, and it is one of the cytokines mainly involved in septic shock [10]. TNF induces vasodilation and enhances the expression of cell adhesion molecules, which facilitates diapedesis and promotes the release of chemokines that recruit neutrophils into the inflammatory site [32,33].

TNF has been one of the most studied cytokines in the course of ASFV in vivo infections. The first study to assess serum concentrations during ASFV infection dates back to 1999. In this study, TNF serum levels rapidly increased in domestic pigs infected with high doses (10^5^ TCID_50_) of the genotype I virulent isolate E75 [34]. Similar results were described soon after in pigs infected with high doses of the genotype I virulent isolate E70 [13]. Increased levels of this cytokine were also observed after infection with virulent ASFV isolates belonging to genotype II, including Armenia07 [16] and SY18 [12]. A small, but statistically significant, rise was observed in pigs infected with the genotype I moderately virulent isolate Netherland’86 [19]. Increased levels of TNF are likely indicative of an overwhelming host inflammatory response, which may contribute to the development of severe lymphopenia and immunosuppression during acute ASF [13,34]. This point will be discussed later on (Section 3).

In contrast, other studies did not report noteworthy changes in TNF serum levels after infections with virulent ASFV isolates belonging to either genotype I (OURT88/1) [35] or genotype II (SY18, HLJ/18) [18,24]. Conflicting results were also obtained from pigs immunized with deletion mutants or naturally attenuated isolates belonging to either genotype I (BeninΔMGF, OURT88/3) [20,35] or genotype II (SY18ΔL7-11) [24], in which changes in TNF serum levels were not remarkable either. These results contrasted with those obtained after the immunization of pigs with the deletion mutant HLJ/18-7GD (genotype II), in which a significant rise in TNF was reported [18]. The factors underlying the observed differences are unknown.

### 2.4. Pro-Th1 Cytokines IL-2 and IL-12

Cellular immunity plays a major role in protection against ASFV, and in particular, Th1 response (mainly led by NK and CD8^+^ cytotoxic T cells) seems crucial [36].

#### 2.4.1. IL-2

IL-2 is a pleiotropic cytokine that promotes, in synergy with IFN-γ, the Th1 response. It was discovered in 1976 as a T cell growth factor [5,37], and it is released mainly by activated CD4^+^ T cells and activated CD8^+^ T cells [38]. IL-2 plays a crucial role in priming and maintaining both Th1 and Th2 differentiation, as well as in expanding cytotoxic T cells. It can also enhance NK cytotoxic activity, as well as promote the differentiation of regulatory T cells (Tregs) [5,38].

Few studies have analyzed serum values of this cytokine during ASFV infection. Most of these studies reported that IL-2 circulating levels were not modulated after immunization with either virulent or attenuated ASFV strains [21,22,24]. In a recent study, increased serum levels of this cytokine in pigs infected with the virulent isolate HLJ/18, but not with its derived attenuated mutant, HLJ/18-7GD, were described [18]. Also recently, a study reported small, but statistically significant, increased levels of this cytokine during ASFV infection, although not in all infected pigs. Specifically, results revealed an increase of IL-2 serum levels at day 7 pi in farm pigs infected with the moderately virulent isolate Estonia2014, but not in SPF pigs [17].

#### 2.4.2. IL-12

IL-12 is a pro-inflammatory cytokine and a member of the IL-12 family, which also includes IL-23, IL-27 and IL-25. It is a heterodimeric cytokine, like the other members of the IL-12 family, composed of two subunits, p35 and p40, the latter shared with IL-23 [10,39]. IL-12 is produced mainly by antigen presenting cells (APCs), especially monocytes and macrophages, in response to microbial pathogens [10,39]. This cytokine plays a pivotal role in host defenses against intracellular pathogens due to its ability to stimulate both innate and adaptive immune cells [40]. IL-12 is a key inducer of cell-mediated immunity via stimulation of Th1 cells. It synergizes with IL-18, TNF and other proinflammatory cytokines in stimulating IFN-γ production by NK cells and cytotoxic T cells [10]. After its release, a positive feedback loop with T cells is created. IL-12 induces IFN-γ production by T cells, which in return trigger further production of IL-12 by APCs, enhancing Th1 differentiation [39]. IL-12 production must be tightly controlled at several levels to avoid the development of an exacerbated pathological immune responses [40].

To date, relatively few studies have investigated ASFV’s impact on serum levels of IL-12. Immunization of domestic pigs with attenuated deletion mutants, such as ASFV-G-Δ9GL/ΔUK or Pret4∆9GL, did not result in significant modulation of circulating levels of this pro-Th1 cytokine [21,22]. No variation in serum values of IL-12 was observed in pigs infected with the moderately virulent isolate Netherland’86 [19] or the attenuated deletion mutant HLJ/18-7GD [18]. No raise in serum values of IL-12 was detected in pigs infected with the virulent HLJ/18. In fact, at 7 days pi (dpi), animals presented values even lower than those pre-immunization [18]. On the contrary, it was reported that IL-12 serum levels started to rise soon after infection with the highly virulent isolate SY18 (at 3 or 5 dpi) and remained relatively high until the end of the experimental study (7–8 dpi) [12]. No significant modulation was instead observed after IM immunization with the attenuated deletion mutant SY18ΔL7-11 [12]. Overall, these data suggest that increased serum levels of IL-12 do not reflect development of a protective cellular-mediated immunity, but they might rather be a sign of a lethally derailed immune response.

### 2.5. Pro-Th2 Cytokines: IL-4 and IL-13

Broadly speaking, IL-4 and IL-13 promote a Th2 response, mainly characterized by antibodies production [5]. In pigs, as well as in humans, theses cytokines are the solely members of the IL-4 superfamily [6]. IL-4 was first identified as a co-mitogen of B-cells [41]. Later, its crucial role in Th2 cell-mediated immunity, IgE class switching in B cells, tissue repair and homeostasis through alternative macrophage activation (M2) was discovered [42]. It was also demonstrated that IL-4 has anti-inflammatory functions [5]. This cytokine is secreted mainly by Th2 cells, mast cells, eosinophils and basophils [42]. IL-13 shares many biological activities with IL-4. IL-13 is able to promote B-cell proliferation and immunoglobulin class switching, as well as to induce anti-inflammatory status in monocytes [43,44].

The role of antibodies in protection against ASFV is controversial. Some studies reported a protective role of the humoral response against this hemorrhagic virus, shown by the survival of animals against challenge with virulent ASFV isolates after the transfer of plasma or colostrum from convalescent sows [36].

So far, no studies have investigated ASFV’s impact on serum levels of IL-13, whereas only a few studies have paid attention to changes in IL-4 serum values. Some of these studies reported that immunization with either virulent or attenuated ASFV strains did not modulate serum levels of this cytokine [20,24,35]. However, recent studies reported small, but statistically significant, increased levels of this cytokine during ASFV infection. Increased IL-4 serum levels were described in pigs infected with the virulent isolate HLJ/18, but not with its derived attenuated mutant HLJ/18-7GD [18]. In another recent publication, serum levels of IL-4 (as well as other pro-inflammatory cytokines) rose at day 7 pi in farm pigs infected with the moderately virulent isolate Estonia2014, which exhibited severe clinical signs of ASF [17]. Results of that study suggested that the increased IL-4 serum levels in farm pigs were probably indicative of a derailed immune response observed after infection [17].

### 2.6. Pro-Th17 Cytokines: IL-17 and IL-23

Th17 response during ASFV infection and the possible differences among strains of different virulence are still unclear. In this part of the review, we will report available knowledge in the literature on two pro-Th17 cytokines during ASFV infection: IL-17 and IL-23.

#### 2.6.1. IL-17

To date, six IL-17 superfamily members have been identified: IL-17A, IL-17B, IL-17C, IL-17D, IL-17E and IL-17F. Among them, IL-17A has been the most studied member [6]. IL-17A, often referred to as IL-17, is a pro-inflammatory cytokine mainly released by the Th subset, Th17 [45]. Not only Th17, but also γδ T-cells have been pointed to as the main sources of this cytokine. Other cells (NK cells, innate lymphoid cells, CD8^+^ cells, neutrophils, microglia and mast cells) are also able to produce IL-17 [46]. IL-17 plays a crucial role in several infectious diseases, autoimmune disorders and cancer. In pigs, as in humans and other veterinary species, this cytokine is involved in many inflammatory diseases [45]. IL-17 interacts with several mediators [e.g., GM-CSF, IFN-γ, IL-22, IL-1β, TNF] to exert its proinflammatory effect [46] and promotes neutrophil recruitment and activation [47]. This cytokine also plays a pivotal role in gut immunity [45].

To date, only some discording studies have investigated the role of this inflammatory cytokine during ASFV infection. In one study, the first in evaluating the evolution of IL-17 levels during ASFV infections, domestic pigs were inoculated by the IM route with 10^4^ TCID_50_/mL of the highly virulent isolate Armenia07. Serum samples were collected daily from day 0 to 7 pi. Increased serum levels of IL-17 were observed from day 1 to 3 pi. Then, serum levels returned to control values at the end of the experiment (7 dpi) [48]. In subsequent studies, IL-17 serum levels were monitored in domestic pigs infected by the intranasal (IN) route with a low dose (10^3.5^ TCID_50_/mL) or a high dose (10^5.5^ TCID_50_/mL) of the moderately virulent isolate Netherlands’86. Results showed that in all tested animals, serum levels of IL-17 remained stable during the whole experimental study (from day 0 to 27 pi) [19]. The differences observed between the two studies are likely related to the virulence of the isolates and the route of infection used: infections with highly virulent ASFV isolates in domestic pigs often result in a systemic pro-inflammatory state [49], with IL-17 being considered as a key pro-inflammatory cytokine, which has also been suggested as a sepsis biomarker in humans [46].

#### 2.6.2. IL-23

IL-23 is a pro-inflammatory cytokine member of the IL-12 family. It is produced mainly by activated DCs and macrophages in response to microbial pathogens [39]. IL-23 is composed of two subunits, p19 and p40, the latter shared with IL-12. Despite belonging to the same family, IL-23 and IL-12 present different biological properties. Thus, IL-12 promotes the development of Th1 cells, as described above (Section 2.4.2), whereas IL-23 is involved in development of Th17 cells in a pro-inflammatory context [39,50]. IL-23 promotes the differentiation of naive CD4^+^ T cells into Th17, especially in the presence of TGF-β and IL-6 and plays a pivotal role in maintenance of these T cell subsets and IL-17 production [47,50].

To date, only one study has evaluated ASFV’s impact on circulating levels of IL-23 during an experimental infection with the virulent isolate Armenia07 alongside IL-17, as described above (Section 2.6.1). In this study, serum levels of IL-23 started to rise early after infection (day 1 pi), peaking at the end of the experimental observation (day 7 pi) [48]. In parallel, authors observed neutrophil recruitments in the lung of infected pigs. These findings suggested that IL-23 promoted Th17 development and activation during ASFV infection [48]. A dysregulation of the Th17 response might contribute to the development of a systemic pro-inflammatory state during acute ASF. Th17 response activation during ASFV infection and the possible differences among strains of diverse virulence remain unclear.

### 2.7. Anti-Inflammatory Cytokines: IL-10 and TGF-β

#### 2.7.1. IL-10

IL-10 is a member of the IL-10 superfamily alongside IL-19, IL-20, IL-22, IL-24 and IL-26 [6]. This cytokine possesses a potent and broad anti-inflammatory activity, dampening the inflammatory response and preventing inflammatory and autoimmune pathologies [51]. IL-10 was identified in 1989, with macrophages, DCs, B cells, NK and different T cells subsets being among their main producers [51,52]. IL-10 promotes the differentiation of IL-10-secreting Treg cells and polarizes macrophages towards an anti-inflammatory phenotype [10]. In pigs, as in humans, it dampens macrophage production of pro-inflammatory cytokines and expression of MHC class II, thus inhibiting antigen presentation [10,53]. IL-10 exerts its anti-inflammatory activity through the binding to the IL-10 receptor (IL-10R) and activation of signal transducer and activator of transcription 3 (STAT3) [52].

Different and contradictory studies have monitored circulating levels of this cytokine during ASFV infections. An increase of IL-10 serum levels was positively correlated with the survival of pigs to infection with the moderately virulent isolate Netherland’86 [19]. However, in other studies, no remarkable changes in IL-10 levels (with the exception of occasional increases in some animals) were reported after immunizations with different attenuated ASFV strains belonging to genotype I (BeninΔMGF, OURT88/3, BeninΔDP148R, OURT88/3D329L) [15,20,35,54,55] and genotype II (HLJ/18-7GD) [18]. Protected pigs immunized with the attenuated strains BeninΔDP148R or OURT88/3D329L did not display either changes in serum IL-10 concentrations after challenge with the genotype I virulent isolates Benin97/1 or OURT88/1 [54,55]. The same results were observed in protected pigs immunized with OURT88/3 or BeninΔMGF, which did not display any change in serum IL-10 levels after challenge with the virulent isolates OURT88/1 or Benin97/1, respectively. In these experiments, only not protected immunized pigs or non-immunized pigs used as controls that developed acute ASF displayed a significant increase in IL-10 levels after challenge [15,35,55]. In other experimental infections carried out in domestic pigs with highly virulent isolates belonging to genotype II (SY18, HLJ/18), increased IL-10 serum levels were also observed [12,18].

In wild boar (11 animals in total) immunized orally with the genotype II attenuated isolate Lv17/WB/Rie1 that survived after exposition to four animals that had been intramuscularly inoculated with the genotype II virulent isolate Armenia07, IL-10 levels remained relatively constant after immunization and after exposure to infected animals. In the same experiment, the control group constituted non-immunized wild boar (11 in total) exposed to two animals that had been intramuscularly inoculated with the genotype II virulent isolate Armenia07. Them all died or were euthanized between day 13 and 15 post exposure to infected animals. The IL-10 levels in control animals were significantly lower before exposure to infected wild boar than after exposure [56].

Therefore, results suggested a negative correlation between secretion of IL-10 and survival, so that animals that succumbed to infection displayed an increase of IL-10 serum values [12,15,55,56]. In concordance with these results, it has recently been described that early after infection with the moderately virulent isolate Estonia2014, IL-10 serum levels were significantly high in domestic pigs, which displayed more severe clinical signs and higher levels of several pro-inflammatory cytokines than SPF pigs [17]. These findings suggested that the high IL-10 circulating values did not reflect a physiologically coordinated immune response, but were likely a sign of a fatally uncontrolled immune response, as recently reviewed [36]. In accordance with this postulate, recent studies have also suggested that higher levels of Tregs cells were correlated with lethality in animals infected with virulent ASFV isolates [57,58,59]. Overall, it seems that the regulatory components of the immune system, such as Tregs and IL-10, might dampen the development of an effective protective response against virulent ASFV isolates. Future studies should focus more deeply on the role played by such regulatory components during ASFV infections and in the course of immunizations with new vaccine candidates.

#### 2.7.2. TGF-β

TGF-β is a multifunctional cytokine belonging to the TGF-β superfamily, which includes three different isoforms (TGF-β1, TGF-β2, TGF-β3), as well as other 27 proteins [6]. TGF-β is produced by different immune cell types, such as lymphocytes, macrophages and DCs, and it is involved in regulation of inflammatory processes. Thus, TGF-β participates in the initiation and resolution of inflammatory responses through the control of differentiation, proliferation, survival and state of activation of different cell subsets [60,61]. It dampens the inflammatory effects of IL-1β, IL-12 and TNF [10]. TGF-β is also a crucial player in peripheral T cell homeostasis, being necessary for both Treg and Th17 differentiation [61,62]. In pigs, as well as in humans, it polarizes macrophages towards an anti-inflammatory phenotype [53,63]. TGF-β counteracts the development of exacerbated pathological immune responses to self or non-harmful antigens, without affecting immune responses to pathogens [61].

Only a few studies have evaluated ASFV’s impact on circulating levels of TGF-β. The levels of this cytokine were monitored at day 0, 7 and 14 after immunization with the attenuated mutant ASFV-G-Δ9GL/ΔUK. Results showed an increase in TGF-β levels after immunization in some of the tested pigs [22]. In another study, a comparative response in pigs of different ages (either 12 or 18 weeks old) to infection with the moderately virulent isolate Netherlands’86 was evaluated. In this study, serum levels of TGF-β remained mostly stable during the whole experiment (from day 0 to 27 pi) [19]. Similar results were later reported in domestic pigs infected with the highly virulent isolate SY18. In this study, TGF-β serum values did not increase during the whole observation period (from day 0 to 8 pi) [12]. Overall, ASFV infection did not significantly modulate circulating levels of this anti-inflammatory cytokine, although small differences have been observed between strains of diverse virulence.

### 2.8. Interferons (IFNs)

Interferons (IFNs) constitute a family of cytokines that play a pivotal role in orchestrating the innate immune response during viral infections. These proteins were first discovered in 1957 [64] and are regarded as the first line of defense in cells during viral infections [65]. To date, three distinct classes of IFNs have been described: Type I, Type II and Type III IFNs [66].

#### 2.8.1. Type I IFNs

After binding to their receptors, type I IFNs elicit a plethora of biological activities, leading to the transcription of genes, called IFN-regulated genes (ISGs), which are a heterogeneous group of proteins that serve different purposes related to direct antiviral defense and immune regulation. ISGs include PRRs (RIG-I, MDA5 and Toll-like receptors), signaling molecules (MYD88 and MAVS), transcription factors (IRFs), members of the signal transducer and activator of transcription family (STATs) and proteins with direct antiviral functions (Mx proteins and IFITs) [67]. ISG products interfere with different stages of viral lifecycles (viral entry, uncoating, transcription, replication or egress) or inhibit viruses by degrading viral RNA and/or blocking translation of viral mRNAs (MOV10, 2′5′ OAS, rNase L, PKR, ZAP) [68]. Considering their crucial role in the fight against viral infections, several porcine viruses, including ASFV, have deployed strategies to overcome type I IFN effects [69,70,71].

Porcine type I IFNs comprise at least 39 functional genes [6]. Type I IFNs include several families (IFN-α, IFN-β, IFN-ε, IFN-ω, IFN-k, IFN-δ and IFN-τ), some of which present different subtypes [66]. In pigs, 17 IFN-α subtypes have been described, presenting different antiviral, anti-proliferative and immunomodulatory properties [66]. On the contrary, only one IFN-β subtype has been identified in this species [6]. Some studies monitored type I IFNs through evaluation of their bioactivity, whereas others monitored serum levels of either IFN-α or IFN-β proteins using ELISA. Details of these research works are summarised in Table 2.

Serum levels of total type I IFN were studied by monitoring its bioactivity [17,70]. In these studies, the presence of biologically active IFN was evaluated using MDBKt2 cells and a chloramphenicol acetyltransferase (CAT) assay [70] or SK6-MxLuc cells [17]. Results showed an increase in type I IFN levels in pigs infected with genotype II highly virulent (Georgia 2007/1, Armenia08) and moderately virulent isolates (Estonia2014), but not in pigs infected with genotype I attenuated isolates (OURT88/3) [17,70]. In parallel, whole blood transcriptomic module analysis revealed high induction of IFN type I genes after Armenia08 infection [17]. The observed increase of type I IFN was not related to protection but was instead associated with a dysregulated fatal immune response. It was suggested that plasmacytoid DCs (pDCs) were likely the major source of this pathologically high type I IFN [70], considering that pDCs are indeed able to produce high levels of these molecules despite the presence of viral type I IFN antagonists [69]. This dysregulated type I IFN response might be linked to the apoptosis of uninfected lymphocytes observed during acute forms of ASF, similar to what has been described for other viruses, such as classical swine fever (CSF) [69]. To date, only one study has investigated pDCs’ interaction with ASFV [70], thus further research should better elucidate ASFV’s interaction with this cell type, as well as which factors promote the development of pathologically high levels of these antiviral cytokines.

In other studies, circulating levels of IFN-α or IFN-β were investigated. Different studies detected increased levels of IFN-α after infections with genotype I virulent isolates, such as OURT88/1 and Benin97/1 [55,59], or after infections with genotype II virulent isolates, including Georgia 2007/1 and SY18 [12,70,72]. In all these studies, IFN-α levels peaked early after infection (1–3 dpi). On the contrary, no increase was observed in pigs immunized with the moderately virulent isolate Netherland’86 [19] or the attenuated isolates Pret4Δ9GL [21], BeninΔMGF [59], OURT88/3 [55,59] and OURT88/3ΔI329L [55].

Both Golding et al. [70] and Karalyan et al. [72] detected increased levels of IFN-β after infections with high doses of genotype II virulent ASFV isolates. In particular, both IFN-α and IFN-β were observed in serum samples from domestic pigs infected with the virulent isolate Georgia 2007/1, which coincided with viraemia [70]. No increase in both IFN-α and IFN-β were described in pigs immunized with the attenuated deletion mutant Pret4∆9GL [21]. Overall, these data suggest that elevated circulating levels of IFN-α and IFN-β are related to the virulence of ASFV isolates and may be directly correlated with viral loads in blood. However, results reported by Li and colleagues (2021) were different. In this case, the infection of pigs with the attenuated deletion mutant ASFV-Δ7R, but not with its virulent parental strain HLJ/18, resulted in increased serum levels of both IFN-α and IFN-β [23]. Nevertheless, that enhancement was likely linked to a peculiar function of the MGF gene (MGF505-7R) in inhibiting the induction of IFN.

#### 2.8.2. Type II IFN

Like humans and mice, porcine type II IFN consists of one molecular species (IFN-γ) [6]. IFN-γ is primarily secreted by CD4^+^ Th1 cells, NK cells and CD8^+^ cytotoxic T cells [73], and it plays a pivotal role in inducing and modulating an array of immune responses. IFN-γ exerts its function through binding of the IFN-γ receptor (IFNGR), which activates a downstream signal transduction cascade, ultimately leading to the regulation of several genes’ expression [73,74]. IFN-γ promotes inflammation, NK cell activity and macrophage classical activation. It also enhances antigen presentation and orchestrates activation of the innate immune system [73,74,75]. This cytokine possesses a strong antiviral activity [65], thus several viruses have evolved multiple IFN-γ escape strategies. It has been described that virulent ASFV isolates have developed mechanisms to efficiently replicate in macrophages stimulated with this anti-viral molecule [36].

Immunization studies in vivo with attenuated ASFV strains revealed that protection against challenges carried out with virulent isolates was correlated with the development of a cellular immune response led mainly by NK and CD8^+^ cytotoxic T cells [36]. A hallmark of NK/cytotoxic T cells activation is the production of IFN-γ; thus several research works assessed the production of this antiviral cytokine by using diverse techniques such as ELISA, ELISpot assay or flow cytometry [36,76].

(a) IFN-γ levels in serum

ELISA techniques were used to quantify circulating levels of IFN-γ in diverse studies (as summarised in Table 3). Increased serum levels of this cytokine were observed in some pigs immunized with attenuated isolates such as BeninΔMGF [15,20,59], BeninΔDP148R [54] or HLJ/18-7GD [18]. Nevertheless, different studies highlighted that IFN-γ secretion was not synonymous with protection, as its levels were not increased in serum samples from pigs immunized with attenuated isolates that survived after challenge with virulent isolates [15,21,22,35] or in pigs that survived after infection with moderately virulent strains [19]. In addition, some research works reported that animals infected with virulent ASFV isolates presented high IFN-γ levels and also succumbed to disease [15,35,72]. It was suggested that increased levels of this cytokine in moribund animals could be the result of an impaired immune response [36].

(b) ASFV-specific IFN-γ T-cell responses

A number of ASFV-specific IFN-γ-producing cells can be monitored using either ELISpot assay or flow cytometry, techniques that can be useful to quantify specific T-cell responses. ELISpot assay to detect ASFV-specific IFN-γ-producing cells has been recently described in detail [76]. This assay is often performed using 96-well plates with peripheral blood mononuclear cells (PBMC) re-stimulated ex vivo with a recall antigen (e.g., virus, peptides) [76]. PBMC from surviving inbred pigs infected with the non-virulent genotype I tissue culture-adapted strain BA71V induced IFN-γ in response to homologous stimulation with ASFV, but not in response to stimulation with heterologous or virulent strains [77]. High numbers of ASFV-specific IFN-γ-producing cells in pigs immunized with the attenuated isolate OURT88/3 and boosted with the virulent isolate OURT88/1 correlated well with protection induced in vivo against homologous and heterologous challenge [78]. Some correlation between virus-specific IFN-γ-producing cells induced after immunization with BeninΔDP148R and protection against the virulent parental Benin97/1 was also observed [54]. In addition, correlation between protection and ASFV-specific IFN-γ-producing cells was recently reported [79]. In detail, the immunization of pigs with the BA71ΔCD2 deletion mutant (developed from the virulent genotype I Badajoz-71) conferred dose-dependent cross-protection against direct-contact challenge with pigs infected with the genotype II virulent isolate Georgia2007/1. Protection was associated with IFNγ-secreting cells, evaluated by ELIspot in PBMC (re-stimulated ex vivo with either BA71ΔCD2 or Georgia2007/1) [79].

Nevertheless, in some studies, high numbers of IFN-γ-producing cells did not always correlate to animal survival after challenge, indicating that induction of IFN-γ-specific T cells is not a sufficient indicator of protection either when virulent challenges were carried out shortly after immunizations [80,81,82] or when they were performed long after immunizations [59], which suggests the existence of other mechanisms, although without excluding this one, that would be involved in the protection afforded by different ASFV attenuated isolates [20,21,22,83]. Adenovirus, alphavirus and vaccinia virus vectors have all been shown to induce effective ASFV antigen-specific responses. However, despite the promising immunogenicity data, they have shown limited protection [84,85]. Pools of peptides representing diverse sets of ASFV proteins were screened using lymphocytes from ASFV-immune pigs that had recovered from infection, and specific secretion of IFN-γ was measured using ELISpot [81]. Group of pigs were immunized with pools comprised of different genes for some of the selected proteins that were vectored by an adenovirus (rAd) prime and modified vaccinia Ankara (MVA) boost [81,84]. Netherton et al. [81] observed that the numbers of ASFV-specific IFN-γ-producing cells in the blood were comparable to those observed in other studies using live attenuated strains [20,22,78], and although this immunization regime did not protect animals from severe disease, prime immunization with pools of rAd induced more robust cellular immune responses than the MVA boost. On the contrary, Goatley et al. [84] were able to identify eight genes that were able to protect pigs against fatal disease induced by the virulent isolate OURT88/1. Using ELISpot techniques, ASFV-specific IFN-γ-producing cells were also detected in pigs after one single immunization either with OURT88/3 or the deletion mutant BeninΔMGF, with a peak at 24 days post-immunization. However, a single dose did not induce long-term protection against challenge [59].

Flow cytometry can be adopted to better characterize ASFV-specific IFN-γ-producing cells. Some studies were only able to speculate on the possible origin of IFN-γ synthesis [54,86,87,88]. In a review, Takamatsu et al. [89] mentioned some unpublished results in which they observed that IFN-γ^+^ lymphocytes from ASFV-stimulated immune PBMC displayed mainly the CD4^+^CD8^+^ T cell phenotype, of which a third displayed the memory helper T cell (CD4^+^CD8^low^) phenotype and the rest, the cytotoxic (CTL) (CD8^high^) phenotype [89]. So far, few pioneering studies have been carried out. Phenotyping of ASFV-specific IFN-γ-producing cells in pigs after immunization with BeninΔMGF and OURT88/3 have been fully characterized [59,82]. In both studies, the authors observed that the source of IFN-γ in both immunized groups originated from different subsets of CD8^+^ T cells, mainly cytotoxic (CD8^high^) T cells, particularly double-positive memory (CD4^+^CD8^+^) T cells, suggesting a possible role of these cells in protection. Moreover, they highlighted the importance of other IFN-γ-secreting cells in these mechanisms, such as γδ-T cells or NK cells [59,82]. Goatley et al. [82] observed how increased numbers of specific IFN-γ^+^ cells evaluated after the inoculation with the attenuated isolate OURT88/3 in both inbred and outbred pigs did not serve as a protection indicator against challenge with the virulent isolate OURT88/1, and suggested a possible role of antibodies in protection against homologous virulent isolates. However, differences in the cellular responses (mainly of CD8^+^ T cells) in outbred pigs that survived a second challenge with the genotype II virulent isolate Georgia 2007/1 were observed [82]. In a recent work, both flow cytometry and gene expression analysis were employed to characterize ASFV-specific cytokine-producing cells in domestic pigs immunized with BA71ΔCD2, a candidate vaccine that conferred dose-dependent cross-protection against direct-contact challenge with pigs infected with the genotype II virulent isolate Georgia2007/1. Researchers reported that intracellular cytokine staining of PBMC from BA71ΔCD2-immunized pigs, stimulated ex vivo with BA71ΔCD2, showed elevated percentages of ASFV-specific IFNγ- and TNF-producing CD4^+^CD8^+^ T cells, a phenotype characteristic of polyfunctional memory T cells [79]. In the same work, researchers employed bulk and single-cell transcriptomics to characterize immune responses against the deleted mutant BA71ΔCD2. Transcriptomics carried out on PBMC showed that BA71ΔCD2 triggered ASFV-specific activation of Th1 and cytotoxic T cells, concomitant with a rapid IFNγ-dependent triggering of an inflammatory response characterized by TNF-producing macrophages [79].

### 2.9. Chemokines

Chemokines are a group of small, secreted proteins (8–15 kD), which possess the ability to mediate selective recruitment of specific cell types in specific tissues [90]. These molecules play important roles in regulating several immunological processes, including immune surveillance, immune system development, priming and regulation [90]. In pigs, different chemokines have been described including 21 CCL, 1 CX3CL, 1 XCL and 16 CXCL. The CCL chemokine superfamily is characterized by significant differences among species, whereas CXL chemokines are better preserved among species. For example, CCL3 and CCL7 are chemokines present in mice and humans but missing in pigs and cows [6]. Several viruses have developed mechanisms to evade chemokine responses in order to manipulate the outcome of infections to their advantage [91].

#### 2.9.1. CXCL Chemokines and ASFV

CXCL8 is the chemokine whose circulating levels have been most studied during in vivo ASFV infections, although with conflicting findings. On the contrary, there is a paucity of information regarding ASFV modulation of circulating levels of other CXCL chemokines, as reported in Table 4. CXCL8 is broadly known as a potent neutrophil chemoattractant, able to promote the recruitment of neutrophils and other granulocytes to the site of infection [90]. In addition, this chemokine triggers neutrophil degranulation and enhancements in their phagocytic functions [10,92]. CXCL8 is released mainly by macrophages, considered its main source, but also by epithelial cells, endothelial cells and airway smooth muscle cells [10,93].

In the first study carried out to evaluate circulating levels of CXCL8 during ASFV infection, results revealed no significant modulation in vivo of this chemokine after infection with either virulent (Benin97/1, Uganda) or attenuated (OURT88/3) ASFV isolates [94]. Similar results were reported in subsequent studies, in which no significant modulation of these chemokine serum levels was observed after infection with attenuated Pret4∆9GL [21], attenuated ASFV-G-Δ9GL/ΔUK [22], moderately virulent Netherland’86 [19], attenuated SY184L7-11 and virulent SY18 [24] or attenuated HLJ/18-7GD and virulent HLJ/18 [18]. On the contrary, other studies described increased serum levels of CXCL8 after infection with genotype II virulent isolates Armenia07 [16], Armenia08 [17] and SY18 [12]. The factors underlying the observed differences are unknown.

CXCL10, or interferon gamma-induced protein 10 (IP-10), is secreted by different cell types, such as monocytes, macrophages, lymphocytes, keratinocytes, fibroblasts and endothelial cells [10,95]. IFN-γ strongly enhances IP-10 production by several cell types. This CXC chemokine exerts its function through binding to chemokine (C-X-C motif) receptor 3 (CXCR3) [95]. CXCL10 is a chemoattractant for white blood immune cells, such as macrophages, monocytes, activated T cell and NK cells to the site of inflammation [10,96]. It also has a potent angiostatic effect [96] and can promote T cell development, activation, trafficking and functions [10,97].

ASFV’s impact on CXCL10 levels has been investigated in a couple of studies, using qPCR (mRNA levels in whole blood) and western blot (protein levels in plasma) [94] or ELISA assays (protein levels in serum) [12]. It was described that circulating levels of this chemokine rise after infection with virulent ASFV isolates belonging either to genotype I (Benin97/1) or II (SY18) [12,94]. A significant increase of CXCL10 mRNA levels in whole blood was also observed after infection with the genotype I attenuated isolate OURT88/3, but at lower levels than observed after infection with the genotype I virulent isolate Benin97/1. This chemokine promotes T lymphocyte activation and priming toward the Th1 phenotype, as previously stated, thus, authors suggested that release of CXCL10 observed during OURT88/3 infection might be beneficial for the host, helping pigs to mount a protective adaptive immune response against the virus. Nevertheless, CXCL10 has also a pro-apoptotic role (it is able to trigger T lymphocyte apoptosis), thus an aberrant release of this chemokine, as observed during infection with highly virulent ASFV isolates, might contribute to apoptosis of bystander non-infected lymphocytes, often described during acute ASFV infections [94].

CXCL12, also known as stromal cell-derived factor 1 (SDF-1), is a strong chemoattractant for lymphocytes. It also induces intracellular actin polymerization in lymphocytes, which is regarded as a prerequisite for cell motility [98]. It was later described that this cytokine plays a crucial role in angiogenesis by recruiting endothelial progenitor cells (EPCs) from the bone marrow, an effect that was mediated by receptor CXCR4 [99]. In addition, CXCL12 enhances endothelial cell barrier integrity [100].

So far, only one study has monitored circulating levels of CXCL12 during ASFV infections. In this study, serum values of CXCL12 in domestic pigs following infection with the highly virulent isolate Georgia 2007/1 (10^4^ HAD_50_/mL), alongside VEGFA and VEGFB serum levels (described later in Section 2.11), were evaluated. Researchers observed a significant increase in serum CXCL12 levels at day 5 and 6 pi (termination of the observation period). Such an increase came later than the increase of VEGFB plasma levels and was detected once vascular damage and hemorrhages were already present in infected pigs. It was suggested that CXCL12 release could be a compensatory mechanism to counteract the increased vascular permeability at final stages of the disease [101].

**Table 4:** Modulation of circulating levels of chemokines in domestic pigs infected with ASFV isolates of diverse virulence.

Chemokine	ASFV Isolate *	Isolate Virulence	Dose/Route of Inoculation	Day Post-Inoculation (dpi) Analyzed	Impact on Cytokine’s Serum Values	Reference
**CXCL8**	OURT88/3	Attenuated	10^4^ TCID_50_, IM	3, 5, 7, 14, 20	Decrease	[94]
	Benin97/1	Highly virulent	10^4^ HAD_50_, IM	3, 5, 7	None	[94]
	Uganda 1965	Highly virulent	10^4^ HAD_50_, IM	3, 5, 7	None	[94]
	Armenia07	Highly virulent	10^4^ HAD_50_, IM	1, 2, 3, 4, 5, 6, 7	Raise	[16]
	Pret4∆9GL *	Attenuated	10^4^ TCID_50_, IM	7, 10, 14	None	[21]
	ASFV-G-Δ9GLΔUK *	Attenuated	10^4^ TCID_50_, IM	7, 14	None	[22]
	Netherland’86	Moderately virulent	2 × 10^3.5^ or 2 × 10^5.5^ TCID_50_, IN	0, 7, 10, 17, 27	None	[19]
	SY18	Highly virulent	10^3^ TCID_50_, IM	0, 1, 2, 3, 4, 5, 6, 7, 8	Raise	[12]
	SY18	Highly virulent	10^3^ TCID_50_, IM	0, 3, 7, 10, 14, 21	None	[24]
	SY18ΔL7-11 *	Attenuated	10^3^ or 10^6^ TCID_50_, IM	0, 3, 7, 10, 14, 21, 28	None	[24]
	Armenia08	Highly virulent	0.6–1.2 × 10^2^ TCID_50_, IM	1, 2, 3, 4, 5, 6, 7	Raise	[17]
	Estonia2014	Moderately virulent	0.6–1.2 × 10^2^ TCID_50_, IM	2, 4, 5, 7, 9, 11, 14	Raise mainly in farm pigs	[17]
	HLJ/18	Highly virulent	10^6^ TCID_50_, IM	7	Decrease	[18]
	HLJ/18-7GD *	Attenuated	10^6^ TCID_50_, IM	0, 4, 7, 14, 20, 28	None	[18]
**CXCL10**	Benin97/1	Virulent	10^4^ HAD_50_, IM	3, 5, 7	Raise	[94]
	SY18	Highly virulent	10^3^ TCID_50_, IM	0, 1, 2, 3, 4, 5, 6, 7, 8	Raise	[12]
**CXCL12**	Georgia 2007/1	Virulent	10^4^ HAD_50_, IM	1, 2, 3, 4, 5, 6	Raise	[101]
**CCL2**	OURT88/3	Attenuated	10^4^ TCID_50_, IM	3, 5, 7, 14, 20	None	[94]
	Benin97/1	Highly virulent	10^4^ HAD_50_, IM	3, 5, 7	Raise	[94]
	Uganda 1965	Highly virulent	10^4^ HAD_50_, IM	3, 5, 7	Raise	[94]
**CCL5**	SY18	Highly virulent	10^3^ TCID_50_, IM	0, 1, 2, 3, 4, 5, 6, 7, 8	Raise	[12]

* Deletion mutant; IM: intramuscular inoculation; IN: intranasal inoculation; TCID: tissue culture infectious dose; HAD: hemadsorption dose.

#### 2.9.2. CCL Chemokines and ASFV

Very few studies have focused on ASFV’s impact on circulating CCL chemokine levels (summarized in Table 4). CCL2, CCL3, CCL4 and CCL5 are potent chemokines which recruit immune cells to the site of inflammation [10]. CCL2, also called monocyte chemoattractant protein 1 (MCP1), is a potent chemoattractant for monocytes [102]. CCL2 also enhances migration of other cell types, including T cells, NK cells and DCs, in response to inflammation [102,103,104]. It exerts its function through the receptor CCR2 [102]. CCL2 is produced by different cell types, including endothelial cells, fibroblasts, epithelial cells and smooth muscle cells, although monocytes/macrophages are the main source of this chemokine [102]. Among macrophages, CCL2 is mainly released by classically activated macrophages (M1) in the framework of a pro-inflammatory response [10].

CCL3 and CCL4 are two chemokines mainly produced by monocytes/macrophages [105]. In particular, they are mainly released by activated macrophages (M1) in the framework of a pro-inflammatory response [10]. They were originally discovered in 1988 as a protein doublet, called “macrophage inflammatory protein-1” (MIP-1) [106], but later biochemical separation and characterization of MIP-1 yielded two distinct proteins: MIP-1α and MIP-1β [105]. Both CCL3 and CCL4 display several inflammatory properties, including chemotaxis of diverse leukocyte types, such monocytes, T cells, NK cells, DCs and granulocytes [107].

CCL5, or the regulated upon activation normal T cell expressed and secreted (RANTES), is another potent inflammatory chemoattractant. It is mainly released by T-cells and monocytes, although other immune cells can express this chemokine [108]. CCL5 can also bind to diverse receptors such as CCR1, CCR3, CCR4 and CCR5, the latter with highest affinity [108]. It promotes recruitment of diverse immune cell types such as T cells, basophils, eosinophils and DCs to the site of inflammation [10]. With the help of other cytokines released by T cells, such as IL-2 and IFN-γ, CCL5 also induces the proliferation and activation of certain NK cells to form CHAK cells (CC-Chemokine-activated killer) [109].

Domestic pigs infected with virulent ASFV isolates belonging to either genotype I (Benin97/1) or genotype X (Uganda) presented elevated plasma levels of CCL2, which were strongly higher than those detected in uninfected control animals or pigs immunized with the attenuated isolate OURT88/3 (genotype I) [94]. Researchers suggested that increased levels of CCL2 would have led to monocytes recruitment into the circulation, with a subsequent increase in the number of cells susceptible to ASFV infection [94]. In the same study, gene expression of CCL2 and other CCL chemokines in whole blood (mRNA levels) was also evaluated. Results showed that infection of virulent isolate Benin97/1 significantly increased expression not only of CCL2, but also of CCL3, CCL4 and CCL5, although the greatest fold increase was observed for CCL2. CCL5 serum levels during ASFV infection in vivo have also been monitored in a recent study. Researchers reported that infection with the genotype II highly virulent isolate SY18 resulted in increased serum values of CCL5 (RANTES) early after infection (2–4 dpi), which remained elevated until the end of the experimental study (7–8 dpi) [12]. Increased CCL5 serum levels were associated with high levels of several pro-inflammatory cytokines and severe ASF clinical signs, thus the authors suggested that high CCL5 serum values could be a hallmark of a lethally uncontrolled immune status [12].

### 2.10. Colony Stimulating Factors

Colony stimulating factors (CSFs) are a group of cytokines essential to both hematopoiesis and immune competence. Family members include macrophage-CSF (M-CSF; CSF1), granulocyte/macrophage-CSF (GM-CSF; CSF2); granulocyte-CSF (G-CSF; CSF3) [5,6].

#### 2.10.1. G-CSF

G-CSF is the major hematopoietic growth factor involved in the control of neutrophil development and supports the maintenance of steady-state neutrophil levels in vivo [110]. In addition, this cytokine enhances several neutrophil effector functions in response to bacterial infection, such as superoxide anion generation and the release of arachidonic acid, as well as the production of leukocyte alkaline phosphatase and myeloperoxidase [111]. In the clinic, G-CSF has been applied in the treatment of various forms of both congenital and acquired neutropenia [112].

To date, only one study has evaluated the fluctuation of G-CSF levels during ASFV infection. Serum levels of this cytokine were assessed during experimental infection with the genotype II virulent isolate Armenia07, alongside IL-17 and IL-23, as above described (Section 2.6.1 and Section 2.6.2). A significant increase in serum levels of G-CSF was observed at day 3 pi in infected pigs, which displayed levels about four times higher than those observed in healthy controls. Afterwards, G-CSF levels decreased until reaching levels equivalent to those detected in control pigs towards the end of the experiment (7 dpi) [48]. In the same study, researchers observed increased serum levels of IL-17 and IL-23, as well as neutrophil recruitment in the lung of infected pigs. The authors observed that the increase in serum levels of G-CSF was secondary to IL-17 increase, and subsequent to both neutropenia and neutrophil recruitment in the tissues. It was suggested that increased levels of G-CSF would have a compensatory nature, stimulating proliferation of new granulocytes.

#### 2.10.2. GM-CSF

GM-CSF is a cytokine that promotes the production, maturation and activation of neutrophils, macrophages and DCs [113]. In addition, it stimulates the proliferation of neutrophils from common myeloid progenitors, directly and synergistically with other hematopoietic growth factors (G-CSF and IL-6) [114]. GM-CSF is produced by diverse cell types, such as macrophages, lymphocytes, fibroblasts, endothelial cells, chondrocytes and tumor cells, in response to several immunogenic stimuli [115]. GM-CSF induces recruitment of cells belonging the innate immune system. Different studies have also suggested that this cytokine could be used as an effective treatment for encephalitis caused by West Nile virus, diverse bacterial infections and viral pneumonia [116].

To date, serum levels of this colony-stimulating factor during ASFV infections have been evaluated only in a couple of studies. In one research work, serum GM-CSF levels were almost undetectable in domestic pigs infected with 10^3^ TCID_50_/mL of the virulent isolate SY18 (genotype II) or its derived mutant SY18ΔL7-11 during the whole observation period (from day 0 to 28 pi) [24]. On the contrary, serum values of this cytokine were increased at day 7 pi in pigs inoculated with the attenuated isolate HLJ/18-7GD [18]. The factors underlying the observed differences are unknown.

### 2.11. Vascular Endothelial Growth Factors

Vascular endothelial growth factors (VEGFs) are member of the Platelet Derived Growth Factor (PDGF) superfamily, which in pigs includes PDGFA, PDGFB, PDGFC, PGF, VEGFA (VEGF), VEGFB, VEGFC and VEGFD [6]. VEGFs play a pivotal role in angiogenesis. The formation of blood vessels from the existing vasculature is an obligate requirement for the development and survival of an organism, and angiogenesis must be tightly coordinated. Insufficient angiogenesis results in ischemic disorders and impaired organ developments, whereas uncontrolled angiogenesis contributes to tumor progression [117]. VEGFs are crucial in the physiological development and maintenance of the vascular and lymphatic systems. These cytokines stimulate endothelial cells to degrade the extracellular matrix, migrate, proliferate and form tubes, as well as promote endothelial cell survival. VEGFs also increase vascular permeability [118].

Hemorrhages of different severity constitute a hallmark of ASF. Depending on the ASF clinical form developed by infected animals (acute or subacute), hemorrhages have been attributed to different mechanisms such as endothelial cells disruption, intravascular coagulation phenomena, vasodilation or increased vascular permeability [26,119]. VEGF is known to increase vascular permeability, thus it is possible that production of this mediator from infected cells might contribute to the vascular changes that characterize ASF. So far, only one study has investigated VEGF’s role in ASFV infection. Domestic pigs inoculated intramuscularly with 10^4^ HAD_50_/mL of the highly virulent isolate Georgia2007/1 (genotype II) showed an increase in serum VEGFB values starting from day 3 pi, which remained elevated until the end of the experimental study (day 6 pi) [101]. Researchers described that macrophages produced nitric oxide and VEGFB in response to ASFV infection, which was followed by an increase in VEGF-mediated endothelial permeability [101]. This preliminary work suggested a role played by VEGF in coagulopathy and vascular lesions observed during acute ASFV.

## 3. The Tissues Perspective: Cytokine Role in ASFV Immunopathogenesis

### 3.1. Lymphoid Depletion and Cell Death

Cells belonging to the mononuclear phagocyte system, mainly monocytes and fixed macrophage populations in different tissues, constitute the main target cells for ASFV replication, while other cell types can also become infected, but mostly in later stages of the disease [26]. Preliminary electron microscopy and immunohistochemical studies, carried out on tissue specimens taken from pigs experimentally infected with virulent ASFV isolates, pointed to cell apoptosis in lymphocyte infiltrates of non-lymphoid organs and lymphoid organs as the cause of lymphoid tissue destruction, and consequently of the lymphopenia and immunosuppression that characterize acute ASF. Ultrastructural studies also described the presence of biosynthetically activated cells/macrophages (infected and non-infected) in nearby areas where an intense apoptosis of lymphocytes was taking place, suggesting that these cells might have an indirect effect on the massive apoptosis of uninfected lymphocytes by the release of chemical mediators [120,121,122,123]. It was also suggested that the degree of apoptosis in the lymphoid organs was related to the amount of cytokines secreted. This fact, in turn, would depend on the number of ASFV infected cells and on the virulence of the isolate involved, which might explain the greater lymphocyte apoptosis induced by highly virulent compared to less virulent isolates [123].

The role of TNF (formerly known as TNF-α) in lymphocyte apoptosis during acute ASF was suggested in subsequent in vitro studies, where TNFs containing supernatants from ASFV-infected cultures induced apoptosis of uninfected lymphocytes [34]. In the same study, domestic pigs experimentally infected by the IM route with a high dose (10^5^ TCID_50_) of the genotype I virulent isolate España-75, displayed an enhanced TNF mRNA expression from day 2 or 3 in different target organs (spleen, liver and mesenteric, submandibular and mediastinal lymph nodes), which was correlated with viral protein expression (p30 and p54). These animals also displayed an increase in serum concentrations associated with the onset of clinical signs. A preliminary immunohistochemical study carried out on frozen tissue sections from infected pigs, whose quantitative results were not shown, also suggested the presence of cells morphologically identified as macrophages secreting TNF from day 2 pi in the spleen (mainly in red pulp) and from day 3 pi in renal lymph nodes (mainly in sinuses and medulla), whose presence was increased on the following day (at day 3 pi in the spleen and at day 4 in renal lymph nodes), decreasing thereafter. On the other hand, the presence of cells immunolabelled against the viral antigen was higher than TNF-secreting cells and persisted for longer period [34].

Later on, systematic immunohistochemical studies were carried out on formalin-fixed paraffin-embedded (FFPE) spleen, lymph nodes (renal and gastrohepatic), palatine tonsils, thymus, liver and kidney tissue specimens taken from young domestic pigs (30 kg at the start of the experiment) intramuscularly inoculated with a high dose (10^5^ HAD_50_) of the genotype I virulent isolate España-70. Three pigs were used as uninfected controls, while another twenty-one animals were euthanized in batches of three between day 1 and 7 pi. Using commercial ELISA kits, levels of IL-1β and TNF were also evaluated in serum samples taken from euthanized pigs at different days after infection. From day 2 pi onwards, serum concentrations of both cytokines increased significantly with respect to the levels shown by uninfected control pigs, reaching a maximum at day 4 pi for IL-1β and at day 6 pi for TNF [13].

Next, we will focus on summarizing the most characteristic histopathological findings and pathogenic mechanisms that were suggested to explain the role played by different cytokines in the onset and evolution of lesions such as lymphoid depletion that affected different lymphoid organs (lymph nodes, spleen, thymus, palatine tonsils), pulmonary oedema, hepatic lesions or renal hemorrhages that were described in these domestic pigs which developed an acute form of the disease.

#### 3.1.1. Spleen and Lymph Nodes (Gastrohepatic and Renal)

Images of cell death by apoptosis, characterized by the presence of pyknotic cells, cell fragmentation and engulfed apoptotic bodies by macrophages (tingible body macrophages) were observed during histopathological evaluations in lymphoid follicles (B-cell area) of the spleen as well as in lymphoid follicles (B-cell area) and diffuse lymphoid tissue (T-cell area) of lymph nodes (gastrohepatic and renal) taken from animals euthanized at day 2 pi [13,14]. Such lesions increased in severity from day 3 pi onwards, displaying, in both the splenic and lymph node follicles, a severe lymphoid depletion. Lymph nodes also displayed a severe lymphoid depletion affecting diffuse lymphoid tissue along with hemorrhages. The appearance and increase of apoptotic phenomena was confirmed “in situ” by terminal deoxynucleotidyl transferase dUTP nick end labelling (TUNEL) technique. These changes were accompanied by a significant increase in the number of SWC3^+^ monocytes–macrophages (m-Mo) in splenic structures (splenic cords, marginal zones and follicles) from day 2 pi and lymph nodes (cortex and medulla) from day 3 pi onwards. The presence of cells immunolabelled against viral antigen (viral protein-Vp73), mainly m-Mo and reticular cells, but not lymphocytes, was observed in splenic cords and marginal zones of the spleen and in the medulla of lymph nodes from day 1 pi. This presence was increased throughout the experiment, especially in splenic cords and the marginal zone of the spleen, as well as in the medulla of lymph nodes, peaking between day 4–5 pi [13,14].

In the cortex of lymph nodes, intra-follicular m-Mo that secreted TNF, IL-1α, IL-1β and IL-6 peaked between day 2 and 4 pi. Except for cells marked against IL-6, which decreased after day 3 pi, the presence of m-Mo immunolabelled against these cytokines remained high within the lymphoid follicles until the end of the experiment at day 7 pi. In the medulla, the evolution of cytokines secretion was similar to that described in the cortex. Capillary endothelial cells labelled against TNF and IL-1α were observed occasionally from day 5 pi onwards. However, the presence intra-follicular m-Mo marked against IL-1α and IL-6 in the spleen was almost negligible, although its number increased progressively until the end of the experiment. Intra-follicular m-Mo immunolabelled against IL-1β and TNF were present in higher number, reaching their highest values between day 5–7 pi and coinciding with the maximum presence of cells marked against IL-1α and IL-6 [13,14].

Taken together, these results suggested that: (i) the increase of m-Mo in the spleen and lymph nodes coincided with the appearance of cells with signs of viral infection, changes that preceded the appearance of lymphocyte apoptosis, lymphoid depletion and hemorrhages; (ii) the infection of m-Mo did not inhibit their biosynthetic activation; (iii) many of m-Mo that secreted cytokines did not display signs of viral infection; (iv) cytokines, especially TNF and IL-1 (α and β) released by m-Mo (infected and non-infected), were pointed out as being responsible for the appearance and severity of lymphoid depletion and hemorrhages observed in the spleen and lymph nodes, while IL-6 would play a role in the modulation of inflammatory changes and immune response only at early stages after ASFV infection. Among these cytokines, other studies focused on understanding hemorrhagic viral diseases mechanisms have also pointed to TNF as a determinant in the apoptosis of different cell populations. In addition, this cytokine is able to stimulate the synthesis of other cytokines, such as IL-1 and IL-6, with overlapping and synergic effects, which would contribute to increasing the severity of tissue damage and apoptosis phenomena resulting from viral infections, leading to a state of immunosuppression [124,125,126,127,128].

The ASFV genome contains genes homologous with proteins known for inhibiting apoptosis [129,130,131]. This fact might contribute to promoting the survival of infected macrophages, which might result in a more efficient and productive infection [123]. In addition, the virus may increase the biosynthetic activation and cytokine secretion of infected macrophages, also contributing to the cytokine secretion by non-infected neighboring cells as a result of a paracrine effect [25]. In later stages after infection, cytopathic effect caused by virus replication (necrosis) would contribute to reducing the number of macrophages observed in the splenic cords, marginal zone of spleen as well as in the cortex and medulla of the lymph nodes. However, it would not explain the severe depletion by lymphocyte apoptosis observed in cells that were not pointed out as target cells of the virus [120,122,132]. Therefore, there was a clear association between the arrival of ASFV to the spleen and lymph nodes, the increase of m-Mo, the secretory activation of many of these cells (infected and non-infected) and the severe lymphoid depletion due to apoptosis, existing differences that would depend on the organ and structure evaluated. These facts suggested that cytokine response would not be the result of a systemic, coordinated and uniform response, but would be triggered by the arrival of the virus to each organ or tissue compartment. As mentioned above, among the cytokines evaluated whose main source were the monocytes–macrophages, TNF was suggested as the principal factor responsible for lymphocyte apoptosis as a result of a paracrine effect. However, the synergistic effect played by others of the cytokines evaluated with apoptotic capacity, such as IL-1β and IL-6, could not be ruled out. Therefore, an aberrant cytokine storm induced by ASFV would be the final factor responsible of the massive lymphocyte apoptosis observed in lymphoid organs from the initial stages after infection. The hypoxia caused by phenomena such as hyperemic splenomegaly or hemorrhagic lymphadenitis may also contribute to a massive destruction of lymphocytes, but by necrosis and only at the final stages of the infection.

#### 3.1.2. Thymus

The thymus did not display any remarkable histopathological changes until day 3 pi, when the occasional presence of pyknotic cells, cell fragmentation and tingible body macrophages containing cell debris in the cytoplasm were observed in the thymic cortex, giving rise to the so-called “starry-sky” appearance. These changes increased over the next days leading to an intense depletion of the cortex in those animals that were euthanized between day 4 to 7 pi. Lymphoid depletion, along with an increase of apoptotic cells identified by TUNEL technique, was also observed in the medulla from day 3 pi onwards. Relevant vascular changes, such as hemorrhages were not observed in any of the thymus samples taken throughout the experiment. Progressive lymphoid depletion was accompanied by a significant increase in the number of SWC3^+^ m-Mo from day 3 pi onwards in the medulla and from day 4 pi onwards in the cortex. The number of SWC3^+^ m-Mo in the thymic cortex was higher than in the medulla from day 4 pi onwards. The presence of cells immunolabelled against viral antigen (Vp73), mainly macrophages and reticulo-epithelial cells, was observed from day 3 pi in the medulla and corticomedullary junction. Thereafter, cells marked against the viral antigen, that also included occasionally lymphocytes, were increased in both the medulla and cortex, peaking between day 5 and 7 pi [14,133].

As described for lymphoid follicles in the spleen and lymph nodes, cells immunolabelled against cytokines increased from day 3 pi in both the thymic cortex and the medulla in parallel to lymphocyte apoptosis. Such cells, mainly macrophages, were observed in nearby areas where lymphocyte destruction was taking place. In the thymic cortex, cells secreting IL-1β and IL-6 were the first to increase from day 3 pi, followed by a significant increase in macrophages immunolabelled against IL-1α from day 5 pi and cells marked against TNF at days 6 and 7 pi. In the medulla, cells that secreted IL-1β, IL-1α and IL-6 were increased from day 3 pi while cells immunolabelled against TNF increased from day 5 pi and peaked at day 6 pi. Again, macrophages immunolabelled against TNF constituted the largest secretory cell population in both the thymic cortex and medulla at the final stages of the experiment (day 6–7 pi) when lymphoid depletion was more severe, showing this cytokine as the main responsible for apoptosis of lymphocytes in the thymus [14].

#### 3.1.3. Palatine Tonsil

From day 3 pi, histopathological changes observed in palatine tonsils were characterized by the presence of lymphoid depletion in lymphoid follicles and interfollicular areas with images of pyknotic cells, cell fragmentation and tingible body macrophages with engulfed cell debris. Vascular changes such as hyperemia and hemorrhages were also described. An increase of TUNEL-positive cells was described from day 3 pi in interfollicular areas and within lymphoid follicles, peaking between day 6 and 7 pi. Viral antigen (Vp73) was detected from day 3 pi in interfollicular areas and from day 4 pi onwards in lymphoid follicles, the marginal zone and tonsillar epithelium. The number of cells marked against the viral antigen, mainly macrophages, increased significantly in interfollicular areas and, to a lesser extent, in lymphoid follicles and the marginal zone between day 5 and 7 pi, not presenting in tonsillar epithelium an increase of positive cells against Vp73, mainly macrophage infiltrates and some epithelial cells. In parallel with lymphoid depletion and apoptosis phenomena, a progressive increase of SWC3^+^ macrophages was observed from day 3 pi onwards in interfollicular areas, lymphoid follicles, the marginal zone and tonsillar epithelium, peaking between day 5 and 7 pi. Such an increase was especially remarkable within the lymphoid follicles. A mild increase in macrophages immunolabelled against IL-1α was observed from day 3 pi in interfollicular areas and the tonsillar epithelium and from day 4 pi onwards in lymphoid follicles, peaking in all areas evaluated between day 5 and 7 pi. Regarding cells secreting TNF, only a few were observed in lymphoid follicles and the marginal zone from day 2 pi, increasing moderately in number between day 4 and 7 pi in parallel with a moderate increase also observed in interfollicular areas. TNF^+^ cells were not detected in the tonsillar epithelium [134].

Therefore, in palatine tonsils, macrophages immunolabelled against IL-1α constituted the largest secretory cell population in lymphoid follicles, the marginal zone and interfollicular areas at the final stages of the experiment (day 5–7 pi) when lymphoid depletion and apoptosis phenomena were more severe. An increase in the number of macrophages in lymphoid structures of the palatine tonsil, many of them secreting pro-apoptotic cytokines, occurred in parallel with the appearance and increase of cells immunolabelled against the viral antigen in the same locations from day 3 pi onwards. Therefore, virus infection of macrophages would trigger the biosynthetic activation of infected and non-infected macrophages, also contributing through different chemical mediators’ release to the recruitment and increase of macrophages, as well as to the massive destruction of non-infected lymphocytes due to a cytokine storm [134].

### 3.2. Liver and Acute Phase Response

Other studies were focused on ascertaining the role played by cytokines secreted by hepatic m-Mo populations in the pathogenic mechanisms of hepatitis that appeared in domestic pigs during acute forms of ASF. From day 3 pi, histopathological studies revealed the presence of vascular changes in portal spaces and interlobular septa (hyperemia and oedema), along with cell infiltrates constituted by neutrophils, lymphocytes and macrophages. In addition, hepatic sinusoids displayed a large number of circulating cells, mainly neutrophils, monocytes and occasional lymphocytes, along with enlarged Kupffer’s cells (KC). These lesions increased in severity from day 5 pi onwards, appearing images of pyknosis and cell fragmentation characteristic of apoptosis among the cell infiltrates, circulating cells and KC. Towards the end of the experiment (day 6 pi), sinusoidal endothelial cells appeared swollen. Viral antigen (Vp73) was detected mainly in the circulating monocytes of sinusoids and KC from day 3 pi onwards. The maximum number of cells with signs of viral infection was detected between day 5 and 6 pi, highlighting the presence of an important number of KC, macrophages present at portal spaces infiltrates and interlobular septa infiltrates, as well as hepatocytes immunolabelled against the viral antigen. From day 1 pi, circulating monocytes SWC3^+^ in sinusoids significantly increased and stayed high in number until the end of the experiment. However, the number of KC and macrophages in portal infiltrates SWC3^+^ suffered a significant increase at later stages between day 3 and 5 pi, declining thereafter. In parallel, a significant increase in sinusoidal circulating monocytes immunolabelled against IL-1α, and, to a lesser extent, against TNF and IL-6, was confirmed from day 1 pi. This increase did not become significant for KC and portal macrophages that secreted IL-1α, TNF and IL-6 until day 3 pi. Sinusoidal circulating monocytes, KC and portal macrophages that secreted IL-1α, TNF and, to a lesser extent, IL-6, peaked at day 5 pi, decreasing progressively at day 6 and 7 pi [135].

These results evidenced an early numerical increase (from day 1 pi) and biosynthetic activation of sinusoidal circulating non-infected monocytes that secreted chemoattractant mediators (mainly IL-1α and TNF) prior to virus detection in the liver, which suggested the existence of a mechanism to recruit target cells susceptible to virus infection. Results also evidenced a numerical increase and biosynthetic activation of the resident populations of infected and non-infected hepatic macrophages, mainly KC, which occurred after the detection of the viral antigen in some of these cells (from day 3 pi onwards) in parallel to the onset of hepatitis. In this sense, IL-6, a chemical mediator that is activated by TNF [136] and that may prompt the appearance of cell infiltrates [137], was suggested as an important modulator of immune responses and inflammatory lesions observed in the liver. Additionally, there was a clear association between the infection of hepatocytes and the numerical increase and biosynthetic activation of hepatic macrophages, mainly KC, which suggested that chemical mediators released by KC might induce the expression of surface receptors required for virus infection and replication in cells not belonging to the mononuclear phagocytic system, such as hepatocytes [135].

Results obtained in this study also suggested a direct association between some of the cytokines secreted by KC and other hepatic macrophages and the serum levels reached by APP synthetized by hepatocytes. In this sense, changes in the number of KC and other hepatic macrophages secreting IL-1α and TNF were linked to serum levels’ evolution of C-reactive protein (CRP) and serum Amyloid A (SAA), while the serum levels of haptoglobin (HP) were associated with the secretion of IL-6 [138].

### 3.3. Lung and Pulmonary Oedema

Previous electron microscopy studies carried out on lung samples from domestic pigs infected with a highly virulent ASFV isolate (Malawi 83) suggested that the biosynthetic activation of pulmonary intravascular macrophages (PIMs) would be involved in the mechanisms responsible for the increase in vascular permeability of pulmonary capillaries and the appearance of pulmonary edema characteristic of acute forms of ASF [139]. In subsequent studies, immunohistochemical techniques were implemented to evaluate virus infection, as well as cytokines secretion by the different pulmonary macrophage populations on lung samples from domestic pigs infected intramuscularly with the genotype I virulent isolate España-70. Morphological evaluations showed a mild septal thickening in lung samples from pigs euthanized at day 3 pi. From day 4 pi, along with more severe septal thickening, animals also displayed mild interstitial edema. Interstitial edema became more severe in subsequent days, with appearing alveolar edema also appearing and lesions that became especially severe at days 6 and 7 pi. Viral antigen (Vp73) was not detected in any of pulmonary macrophages until day 3 pi. However, although the number of PIMs immunolabelled against the viral antigen increased as the disease progressed, peaking at day 6 and 7 pi, the number of pulmonary alveolar macrophages (PAMs) remained without remarkable changes. From day 1 pi there was a significant increase in the number of PIMs marked against SWC3, that peaked at day 3 pi and remained high until the end of the experiment. Such an increase was much more moderate and came later (from day 3 pi) in the case of PAMs immunolabelled against SWC3 [140].

PIMs marked against TNF and IL-1α also displayed a significant increase from day 1 pi that peaked at day 3 pi. The presence of a high number of PIMs, mainly secreting IL-1α and, to a lesser extent, TNF, remained high in subsequent days until the end of the experiment. On the contrary, the number of PAMs that secreted cytokines did not display relevant changes over the course of the experiment. Only from day 2 pi, the occasional presence of PAMs marked against TNF (which were not observed in non-infected control pigs) was described, without remarkable changes in their number over the following days. Regarding PAMs marked against IL-1α, lung samples from infected pigs displayed the same number as non-infected control pigs [140]. The early increase of PIMs secreting IL-1α and TNF occurred before ASFV detection in the lung, suggesting that it was induced by circulating chemotactic mediators released by other activated cells from different virus target organs. Both IL-1α and TNF have marked chemotactic activity, so they would contribute to attracting and activating more inflammatory cells, as well as to increase lung capillary endothelial permeability, changes that would contribute to the early appearance of a mild interstitial edema and septal thickening. However, the highest number of PIMs secreting IL-1α and TNF occurred just after the viral antigen was detected in this pulmonary macrophage population from day 3 pi. From that moment, there was a significant increase in the number of PIMs (infected and not infected) secreting cytokines, especially IL-1α, and to a lesser extent, TNF, in parallel to the appearance of the most severe pulmonary lesions, including cell infiltrates in lung septa, as well as severe interstitial and alveolar edema. Therefore, contrary to the role played by PAMs, the biosynthetic activation of PIMs was key in the induction of lung inflammatory changes during acute ASF [140].

### 3.4. Kidney and Hemorrhages

Preliminary electron microscopy studies carried out on kidney samples taken at the middle stage of ASF acute forms (day 5 pi) from pigs infected with a virulent isolate (Malawi 83; genotype I) revealed no evidence of virus infection or replication in the endothelial cells of interstitial capillaries despite the existence of interstitial hemorrhages. These findings ruled out virus replication and destruction of endothelial cells as the initial cause of renal hemorrhages. However, the virus could contribute to the appearance of renal hemorrhagic lesions at the final stages of acute forms when virus replication was observed within renal capillary endothelium. When the presence of renal interstitial hemorrhages was described at the initial stages of the disease, non-infected capillary endothelial cells displayed ultrastructural changes in phagocytic activation. It was suggested that such activation, characterized by endothelial cells’ hypertrophy, occurred to ensure that cell debris from other injured tissues were removed from the blood circulation. Endothelial activation caused the loss of endothelial cell junctions along with a progressive endothelial disruption that caused a generalized detachment of the endothelium, giving rise to erythrocytes and cell debris to move into the interstitium, causing hemorrhages. Endothelial activation and hypertrophy also coincided with an increase in the number of nearby macrophages, with and without signs of viral infection, which also displayed the ultrastructural changes characteristic of biosynthetic activation. These changes led to the hypothesis that chemical mediators released by activated macrophages could also contribute to endothelial activation and disruption [141].

Histopathological and immunohistochemical studies were subsequently carried out on kidney samples taken from domestic pigs infected intramuscularly with the genotype I virulent isolate España-70 that were euthanized sequentially between day 1 and 7 pi [142,143]. As in previous studies, histopathological evaluation did not reveal the presence of remarkable renal lesions up to day 5 pi. Lesions were characterized by their presence in both the renal cortex and medulla of mild and occasional interstitial hemorrhages and edemas, along with mild interstitial infiltrates constituted by enlarged macrophages. The most severe lesions were described at day 7 pi. Such lesions were characterized by the presence of cell debris and fibrin deposits able to obliterate glomerular capillaries, which also displayed swollen endothelial cells and enlarged circulating monocytes. A diffuse congestion of interstitial capillaries of the renal cortex and medulla, along with a high number of enlarged circulating monocytes, neutrophils, lymphocytes, cells debris and fibrillar materials identified as fibrin were also described. In addition, interstitial capillary endothelial cells were hypertrophic and contained intracytoplasmic engulfed cell debris. Interstitial edema was more severe and diffuse than observed in previous days, especially in the medulla. The presence of small, scattered interstitial hemorrhages and interstitial mononuclear infiltrates was also more frequent in both the renal cortex and medulla than in previous days. Infiltrates mainly constituted large macrophages, along with some lymphocytes and occasional plasma cells. Cell death phenomena among cells of the infiltrates were quite intense, with many hypertrophic macrophages displaying intracytoplasmic engulfed cell debris. From day 3 pi, viral antigen (Vp73) was detected in few circulating monocytes present within the glomerular capillaries. However, until day 5 pi, the viral antigen was not detected in any renal structure. From this day, along with a significant increase in monocytes with signs of viral infection within glomerular capillaries, the viral antigen was also detected in circulating monocytes within interstitial capillaries, as well as in macrophages of the interstitial infiltrates present in the cortex and medulla. The number of these cells immunolabelled against the viral antigen increased as the disease progressed. Only at day 7 pi, endothelial cells of some interstitial capillaries located in the cortex and medulla appear to be immunolabelled against the viral antigen. In parallel with the detection of the first circulating infected cells at day 3 pi, in both interstitial and glomerular capillaries, as well as in the interstitium, there was an increase in the number of cells marked against SWC3 (monocytes-macrophages). However, only in the interstitium was the increase of SWC3 positive cells significant from day 5 pi onwards, continuing until the end of the experiment at day 7 pi [142,143].

Additionally, in parallel to the detection of the first infected cells in the kidney, there was an increase in the number of monocytes and macrophages immunolabelled against TNF, IL-1α and IL-6, cytokines with a well-known synergic effect on the vascular system [144]. Such an increase was significant from day 5 pi, peaking at day 6 pi and decreasing slightly at day 7 pi. This highlighted the presence of numerous scattered interstitial macrophages immunolabelled against TNF, IL-1α and IL-6 in the cortex and medulla, as well as the presence of clusters of immunolabelled cells in the nearby of interstitial capillaries. The presence of a lower but important number of circulating monocytes immunolabelled against TNF and IL-1α within interstitial capillaries was also remarkable, with these marked cells being frequently observed in close contact with capillaries endothelial cells. Additionally, occasionally at day 6 and 7 pi, interstitial capillaries’ endothelial cells appeared immunolabelled against TNF and IL-6. Therefore, these results would confirm that when renal hemorrhages were present (day 5 pi), interstitial capillaries’ endothelial cells did not display any sign of viral infection, ruling out virus replication in endothelial cells as the main cause of hemorrhages and edemas. Results would also confirm the role played by cytokines released by activated macrophages (infected and non-infected) in the nearby interstitial capillaries in the mechanisms involved in the activation and disruption of endothelial cells, as well as in the appearance of interstitial hemorrhages and edemas, relegating to a secondary and late role the direct action of ASFV [142,143].

### 3.5. Cytokines Gene Expression after Immunization or Infection with ASFV

Other studies carried out over the course of infections (natural or experimental) or immunizations with vaccine candidates against ASFV have also implemented molecular and/or immunological techniques to evaluate cytokines in tissue samples and their role in the mechanisms of immune regulation and immune protection.

In order to understand the cooperative mechanisms and the response processes of various tissues in pigs after ASFV infection, a retrospective study was carried out in tissue samples (including the lung, spleen, liver, kidney and lymph nodes) collected in China from three pigs found dead in the field, where the upregulation and downregulation of genes was evaluated using transcriptomic and proteomic analyses. The genotype II virulent SY-18 isolate was identified by qPCR as responsible for the pigs’ disease. The results showed that pig tissues cooperated in response to ASFV infection and coordinated a defense against the virus in the form of an inflammatory cytokine storm and interferon activation (type I and III). After virus infection, results suggested an important role in the response of lungs, lymph nodes and the spleen, mainly focused on the innate immune response pathway and energy metabolism regulation, while the liver and kidney were focused on the metabolic regulatory pathway and the inflammatory response [145].

On the other hand, to evaluate the role played by cytokines in the immune-protective mechanisms induced by the multigene family (MGF) and CD2v gene-deleted ASF vaccine candidate HLJ/18-7GD (constructed from the highly virulent ASFV strain HLJ/18 of genotype II), the relative gene expression of a panel of cytokines (IL-1α, IL-1β, IL-6, TNF, IL-12, IL-18, IFN-γ, IL-4, IL-10 and IFN-α) was evaluated by RT-qPCR in different tissue samples. Tissue samples (spleen, tonsils, thymus, submaxillary, gastrohepatic and mediastinal lymph nodes) were taken at day 28 post-immunization from SPF pigs (6–7 weeks old) intramuscularly immunized with a dose of 10^6^ TCID_50_. The same analysis was also performed on tissue samples taken from non-immunized control pigs. Results revealed that the relative gene expression of some of these cytokines (IFN- γ, IL-1α, IL-1β, IL-6, IL-10, IL-12 and IL-18) was upregulated in the lymph nodes of immunized pigs compared to those taken from non-immunized control animals. However, the relative gene expression of TNF was upregulated in all lymphoid organs of non-immunized control pigs regarding pigs immunized with HLJ/18-7GD. It was suggested that the reactivity and lymphocytic proliferation observed in the lymph nodes of immunized pigs would be linked to the upregulation of the cytokines’ gene expression observed, pointing to HLJ/18-7GD as an effective vaccine candidate able to enhance immune-protective mechanisms regulated throughout these cytokines [18].

RT-qPCR was also employed by Lacasta and colleagues to investigate the expression of cytokines and other key immune genes representative of Th1, Th2 and Th17 responses in the gastrohepatic lymph nodes of ASFV-infected pigs. In detail, researchers investigated the expression of these genes in animals infected either with the virulent E75, or its derived culture adapted strain E75CV1, alongside control pigs [88]. At 1 dpi, ten genes were significantly modulated in gastrohepatic lymph nodes of E75CV1-infected pigs, whereas only four genes were upregulated in the group of pigs infected with E75 isolate. At 7 dpi, ten genes were modulated in the E75CV1-group: six upregulated (IFN-γ, IL-5, TNF, TGF-βR1, IL-21, IL-23) and four downregulated (DEFβ2, CD163, IL-13, IL-18). Few genes were modulated at 31 dpi in gastrohepatic lymph nodes from animals that survived after infection with the culture-adapted strain E75CV1: three upregulated (IFN-γ, IL-23, NFκβ) and two downregulated (IL-1β, IL-4). On the contrary, at 7 dpi, several pro-inflammatory cytokines were fiercely upregulated in organs of the E75-infected pigs (IL5, IL-6, IL-8, IL-1β, IL-21, IL-23), as well as IL-10 and two other key immune genes (DEFβ2, TLR3). This unbalanced immunological activation was related to severe ASF clinical signs and preceded the fatal outcome of animals infected with this virulent isolate [88].

Upregulation of pro-inflammatory cytokine genes was also observed in the spleen of pigs infected with high doses of the genotype IX virulent isolate Ken12/busia.1 [146]. In detail, groups of pigs were immunized with high (10^6^), medium (10^4^) or low doses (10^2^) of Ken12/busia.1 alongside an uninfected control group, and clinical signs were monitored until day 29 pi. Pigs belonging to the high-dose or medium-dose group displayed ASF clinical signs and were euthanized once they reached a humane endpoint, between day 7 and 17 pi. On the contrary, pigs belonging to the low-dose group and control pigs did not show ASF clinical signs and were killed at the end of the experiment (29 dpi). Spleens were collected after death and RNA-seq analysis revealed a marked association with severe ASF pathogenesis (especially in the high-dose group) with the upregulation of several pro-inflammatory interleukins (especially IL-6 and IL-17), chemokines (CCL2, CCL4, CXCL2, CXCL10) and VEGFA [146]. These results support the hypothesis that the massive release of pro-inflammatory cytokines is not a sign of a coordinated immune response, but rather evidence of an aberrant “cytokine storm” which is related to ASF pathogenesis.

In other recent study, a recombinant ASFV mutant, ASFV-D9L/D7R, bearing combinational deletions of MGF360-9L and MGF505-7R, was constructed from the genotype II virulent isolate CN/GS/2018 currently circulating in China. One-month-old Large White-Duroc crossbred pigs immunized intramuscularly with a high dose of 10^4^ HAD_50_ of the mutant remained clinically healthy without any serious side effects. During the challenge with a lethal dose (10^2^ HAD_50_) of the homologous virulent isolate CN/GS/2018, vaccinated pigs (5/6) were protected and clinical indicators tended to be normal until the end of the experiment at day 18 post-challenge (pc), while all within-pen contact pigs (4/4) displayed clinical signs and pathological findings consistent with ASF, being euthanized between day 7–8 pc. Results revealed that the two deletions in the mutant not only synergized in boosting a more pronounced interferon response and higher expression of inflammatory and innate immune genes ex vivo, but also elicited an ASFV-specific IFN-γ response together with a p30-specific IgG response, which coincided with protective efficacy. Using immunohistochemical techniques on spleen samples taken at study termination from both protected immunized and non-protected pigs, T-cell responses were also evaluated in vivo. Results revealed a strong IFN-γ immunostaining in spleen samples from immunized animals that survived until the end of the experiment, along with an increased frequency of CD4^+^ T cells coupled with high levels of CD163^+^ infiltration of macrophages. Instead, the number of cells immunolabelled against these immunological markers was much lower in spleen samples from non-protected contact pigs [147].

Finally, in a recent published study Bosch and colleagues employed single cell RNA-sequencing (scRNA-seq) to characterize immune responses against the deleted mutant BA71ΔCD2. This candidate vaccine conferred dose-dependent cross-protection against direct-contact challenge with pigs infected with the genotype II virulent isolate Georgia2007/1 and that protection was associated with ASFV-specific IFNγ-secreting cells (as reported in Section 2.8.2 (b) of this review). Data from lymph node cells revealed that genes upregulated in all cell subsets were enriched in terms related to IFN-I and IFN-γ responses. Several cell populations were significantly overrepresented in lymph node cells from the vaccinated pig Interestingly, the proinflammatory CXCL10 chemokine was strongly upregulated in plasmablasts, in the undefined CD4^+^ T cell subset and in cross-presenting DCs, thus further validating the induction of a Th1-biased recall response. In the sample from the vaccinated pig, cytotoxic T lymphocytes showed downregulation of CCR7 and CXCR4, a hallmark of differentiation to effector CD8 T cells [79].

## 4. General Summary and Main Conclusions

Overall, this review summarizes the current knowledge of ASFV’s impact on different cytokines levels, either at systemic or tissue level. Infection with virulent ASFV isolates often results in an exacerbated immune response, associated with elevated serum levels of pro-inflammatory cytokines (IL-1α, IL-1β, IL-6, TNF, CCL2, CCL5 and CXCL10) [12,13,16,17,18,34,94].

Increased levels of IL-1Ra, a receptor antagonist released to mitigate the pro-inflammatory effect of IL-1, concomitant with low serum values of different pro-inflammatory cytokines (IL-1α, IL-1β, IL-2, IL-6, IL-18, CXCL8) correlated with reduced mortality after infection with moderately virulent ASFV isolates [17,18,24]. These results suggest that IL-1Ra may play an important role in the immunopathology of ASF and point to the importance of monitoring the IL-1Ra/IL-1 ratio during experimental studies with ASFV.

Increased serum values of both type I IFN [12,17,55,70,72] and IL-10 [12,15,17,55,56,59] were also observed in domestic pigs infected with virulent ASFV isolates and seem to be negatively correlated with animal survival.

Different studies also suggested that increased circulating levels of IFN-γ were not correlated with protection of domestic pigs [15,19,21,22,35,59,72]. On the contrary, correlation between virus-specific IFN-γ-producing cells and pig protection was described in other studies [54,77,78,79]. It has been also suggested that IFNγ-dependent activation of innate immunity during the recall response to ASFV, as well as a broad cytotoxic response during the first hours of infection, are critical immune components for protection against ASFV [79]. Future studies should elucidate whether protection is related to IFN-γ release by specific cell types as well as the possible role of other immunomodulatory cytokines.

Among the cytokines evaluated in lymphoid tissues during acute ASF, whose main source was monocyte–macrophages, in most of the lymphoid structures evaluated, TNF was pointed to as the main factor responsible for lymphocyte apoptosis, with IL-1β and IL-6 also playing a critical role by inducing a synergistic effect [13,14,134]. These cytokines would also be involved in the mechanisms responsible for inflammatory lesions in the liver [135] and kidneys [143], as well as in the alteration of vascular permeability responsible for pulmonary edema [140] and the disruption of endothelial cells as a cause of interstitial hemorrhages in kidney and lymph nodes [13,14,143].

The “out of control” cytokine release, also called “cytokine storm”, has been described in other diseases, including hemorrhagic fevers such as caused by viruses such as Marburg, Ebola, Yellow fever, Lassa or Dengue, and was associated with threatening damage to tissues and organs [148,149]. A positive feedback loop between cytokine release and cell death was also described, with cytokine release triggering inflammatory cell death with subsequent further pathogenic cytokine production [148]. Similar mechanisms might underlie the “out of control” cytokine storm during acute ASFV infection. In this scenario, treatments finalized at dampening the pro-inflammatory signal might improve clinical outcomes [148].

Finally, analyzing temporal events in infected tissues during natural transmission with virulent and attenuated strains, and in immune and non-immune animals, is critical to understanding disease pathogenesis and immune response. The ASF research community should take advantage of recent technological advances (e.g., genomics, proteomics, transcriptomic) to better understand the immunopathogenic mechanisms of ASFV in order to generate information that will help the global fight against this devastating virus through the development of new vaccines or treatments.

## Figures and Tables

**Table 2:** Modulation of serum levels of type I IFN in domestic pigs infected with ASFV strains of diverse virulence.

Type I IFN	ASFV Isolate *	Dose/Route of Inoculation	Challenge with Different Isolate	Day Post-Inoculation (dpi) or Post-Challenge (dpc) Analyzed	Impact on Cytokine’s Serum Values	Test Used	Reference
**Total type I IFN**	OURT88/3 (attenuated)	10^4^ TCID_50_, IM	-	0, 2, 3, 4, 5, 7, 8, 10 dpi	None	CAT assay, MDBK cells	[70]
	OURT88/1 (highly virulent)	10^4^ HAD_50_, IM	-	0, 1, 2, 3, 5 dpi	Raise	CAT assay, MDBK cells	[70]
	Georgia 2007/1 (highly virulent)	10^4^ HAD_50_, IM	-	0, 1, 2, 3, 4, 5, 7, 8 dpi	Raise	CAT assay, MDBK cells	[70]
	Armenia08 (highly virulent)	0.6–1.2 × 10^2^ TCID_50_, IM	-	1, 2, 3, 4, 5, 6, 7 dpi	Raise	Luciferase assay; SK6-MxLuc cells	[17]
	Estonia2014 (moderat. virulent)	0.6–1.2 × 10^2^ TCID_50_, IM	-	2, 4, 5, 7, 9, 11, 14 dpi	Raise, mainly in SPF pigs	Luciferase assay; SK6-MxLuc cells	[17]
**IFN-α**	Genotype II isolate (highly virulent)	10^4^ HAD_50_, IM	-	1, 2, 3, 4, 5, 7 dpi	Raise	ELISA	[72]
	Georgia 2007/1 (highly virulent)	10^4^ HAD_50_, IM	-	0, 1, 2, 3, 4, 5, 7, 8 dpi	Raise	ELISA	[70]
	Pret4∆9GL * (attenuated)	10^4^ TCID_50_, IM	-	0, 7, 10, 14 dpi	None	ELISA	[21]
	Netherland’86 (moderat. virulent)	2 × 10^3.5^ or 2 × 10^5.5^ TCID_50_, IN	-	0, 7, 10, 17, 27 dpi	None	ELISA	[19]
	OURT88/3 (attenuated)	10^4^ TCID_50_, IM	Benin97/1 (virulent), 10^4^, IM	0, 4 dpi; 3, termination (4 or 5) dpc	Mild increase only post-challenge	ELISA	[59]
	BeninΔMGF * (attenuated)	10^4^ TCID_50_, IM	Benin97/1 (virulent), 10^4^, IM	0, 4 dpi; 3, termination (6–11) dpc	Raise at 4 dpi; raise post-challenge	ELISA	[59]
	Benin97/1 (highly virulent)	10^4^ TCID_50_, IM	-	3, 4 or 5 dpi	Raise	ELISA	[59]
	OURT88/3 (attenuated)	10^4^ TCID_50_, IM	OURT88/1 (virulent), 10^4^, IM	0, 3, 4, 7, 10, 14, 20 dpi; 0, 3, 5 dpc	None post-infection; mild increase post-challenge	ELISA	[55]
	OURT 88/3ΔI329L * (attenuated)	10^4^ TCID_50_, IM	OURT88/1 (virulent), 10^4^, IM	0, 3, 4, 7, 10, 14, 20 dpi; 0, 3, 4 dpc	None post-infection; mild increase post-challenge	ELISA	[55]
	OURT88/1 (highly virulent)	10^4^ TCID_50_, IM	-	0, 3, 4 dpi	Raise	ELISA	[55]
	SY18 (highly virulent)	10^3^ TCID_50_, IM	-	0, 1, 2, 3, 4, 5, 6, 7, 8 dpi	Raise	ELISA	[12]
	HLJ/18 (highly virulent)	10^3^ HAD_50_, IM	-	1, 5, 8 dpi	None	ELISA	[23]
	ASFV-Δ7R * (attenuated)	10^3^ or 10^5^ TCID_50_, IM	-	1, 5, 8 dpi	Raise	ELISA	[23]
**IFN-β**	Genotype II isolate (highly virulent)	10^4^ HAD_50_, IM	-	1, 2, 3, 4, 5, 7 dpi	Raise	ELISA	[72]
	Pret4∆9GL * (attenuated)	10^4^ TCID_50_, IM	-	0, 7, 10, 14 dpi	None	ELISA	[21]
	Georgia 2007/1 (highly virulent)	10^4^ HAD_50_, IM	-	0, 1, 2, 3, 4, 5, 7, 8 dpi	Raise	ELISA	[70]
	HLJ/18 (highly virulent)	10^3^ HAD_50_, IM	-	1, 5, 8 dpi	None	ELISA	[23]
	ASFV-Δ7R * (attenuated)	10^3^ or 10^5^ TCID_50_, IM	-	1, 5, 8 dpi	Raise	ELISA	[23]

* Deletion mutant; dpi: day post-inoculation; dpc: day post-challenge; IM: intramuscular inoculation; IN: intranasal inoculation; TCID: tissue culture infectious dose; HAD: hemadsorption dose.

**Table 3:** Modulation of serum levels of type II IFN in domestic pigs infected with ASFV strains of diverse virulence.

ASFV Isolate *	Dose/Route of Inoculation	Challenge with Different Isolate	Dose/Route of Infection	Day Post-Inoculation (dpi) or Post-Challenge (dpc) Analyzed	Impact on Cytokine’s Serum Values	Reference
Genotype II isolate (highly virulent)	10^4^ HAD_50_, IM	-	-	1, 2, 3, 4, 5, 7 dpi	Raise	[72]
OURT88/3 (attenuated)	10^4^ TCID_50_, IM; (boost 25 dpi 10^4^ TCID_50_, IM)	Benin97/1 (virulent)	10^4^ TCID_50_, IM	0, 3, 5, 7, 10, 20, 28, 32, 46 dpi; 3, 7 dpc	Raise post-challenge in 1 non-protected pig	[20]
BeninΔMGF * (attenuated)	10^2^ TCID_50_, IM; (boost 25 dpi 10^4^ TCID_50_, IM)	Benin97/1 (virulent)	10^4^ TCID_50_, IM	0, 3, 5, 7, 10, 20, 28, 32, 46 dpi; 3, 7 dpc	Mild increase at 5 and 7 dpi in 4/5 pigs	[20]
Benin97/1 (highly virulent)	10^4^ TCID_50_, IM	-	-	0, 3, 7 dpi	None	[20]
Pret4∆9GL * (attenuated)	10^4^ TCID_50_, IM	-	-	0, 7, 10, 14 dpi	None	[21]
ASFV-G-Δ9GLΔUK *	10^4^ TCID_50_, IM	-	-	7, 14 dpi	None	[22]
Netherland’86 (moderately virulent)	2 × 10^3.5^ or 2 × 10^5.5^ TCID_50_, IN	-	-	0, 7, 10, 17, 27 dpi	None	[19]
BeninΔDP148R (attenuated)	10^3^ HAD_50_, IM; (boost 21 dpi 10^3^ HAD_50_, IM)	Benin97/1 (virulent)	10^4^ TCID_50_, IM	0, 2, 4, 7, 10, 15, 37 dpi; 3, 5 dpc	Increase at 4 and 7 dpi	[54]
OURT88/3 (attenuated)	10^3^ or 10^4^ or 10^5^ TCID_50_, IN	OURT 88/1 (virulent)	10^4^ TCID_50_, IM	0, 3, 5, 7, 14, 21 dpi; 3, 5, 7, 14, 19 dpc	Increase post-challenge in non-protected pigs	[35]
OURT88/3 (attenuated)	10^3^ or 10^4^ or 10^5^ TCID_50_, IM	OURT 88/1 (virulent)	10^4^ TCID_50_, IM	0, 3, 5, 7, 14, 21 dpi; 3, 5, 7, 14, 19 dpc	Increase post-challenge in non-protected pigs	[35]
OURT88/1 (virulent)	10^4^ TCID_50_, IM	-	-	0, 3, 5 dpi	None	[35]
Benin∆MFG * (attenuated)	10^3^ TCID_50_, IM; (boost 21 dpi 10^3^ TCID_50_, IM)	Benin97/1 (virulent)	10^4^ TCID_50_, IM	0, 2, 4, 7, 10, 15, 21, 24, 28, 39 dpi; 3, 5, 7, 9, 12, 14, 25 dpc	Raise 4 dpi; post-challenge higher levels in non-protected pigs compared to protected animals	[15]
Benin∆MFG * (attenuated)	10^3^ TCID_50_, IN (boost 21 dpi 10^3^ TCID_50_, IN)	Benin97/1 (virulent)	10^4^ TCID_50_, IM	0, 2, 4, 7, 10, 15, 21, 24, 28, 39 dpi; 3, 5, 7, 9, 12, 14, 25 dpc	Mild raise at 4 and 7 dpi; post-challenge higher levels in non-protected pigs compared to protected animals	[15]
Benin97/1 (highly virulent)	10^4^ TCID_50_, IM	-	-	0, 3, 5 dpi	Mild raise	[15]
OURT88/3 (attenuated)	10^4^ TCID_50_, IM	Benin97/1 (virulent)	10^4^ TCID_50_, IM	0, 4, 24 dpi; 3, termination (4 or 5) dpc	Increase only post-challenge in non-protected pigs	[59]
BeninΔMGF * (attenuated)	10^4^ TCID_50_, IM	Benin97/1 (virulent)	10^4^ TCID_50_, IM	0, 4, 24 dpi; 3, termination (6–11) dpc	Mild increase post-challenge in non-protected pigs	[59]
Benin97/1 (highly virulent)	10^4^ TCID_50_, IM	-		3, 4 or 5 dpi	None	[59]
SY18 (highly virulent)	10^3^ TCID_50_, IM	-	-	0, 1, 2, 3, 4, 5, 6, 7, 8 dpi	None	[12]
HLJ/18 (highly virulent)	10^6^ TCID_50_, IM	-	-	7 dpi	None	[18]
HLJ/18-7GD * (attenuated)	10^6^ TCID_50_, IM	-	-	0, 4, 7, 14, 20, 28 dpi	Raise	[18]

* Deletion mutant; dpi: day post-inoculation; dpc: day post-challenge; IM: intramuscular inoculation; IN: intranasal inoculation; TCID: tissue culture infectious dose; HAD: hemadsorption dose.

## References

[1] Alonso C., Borca M., Dixon L., Revilla Y., Rodriguez F., Escribano J.M. (2018). Ictv Report Consortium. ICTV Virus Taxonomy Profile: Asfarviridae. J. Gen. Virol..

[2] World Organization for Animal Health (WOAH) African Swine Fever (ASF). https://www.woah.org/app/uploads/2022/10/asf-report22.pdf.

[3] Ward M.P., Tian K., Nowotny N. (2021). African Swine Fever, the forgotten pandemic. Transbound. Emerg. Dis..

[4] Dixon L.K., Stahl K., Jori F., Vial L., Pfeiffer D.U. (2020). African swine fever epidemiology and control. Annu. Rev. Anim. Biosci..

[5] Dinarello C.A. (2007). Historical insights into cytokines. Eur. J. Immunol..

[6] Dawson H.D., Sang Y., Lunney J.K. (2020). Porcine cytokines, chemokines and growth factors: 2019 update. Res. Vet. Sci..

[7] Garlanda C., Dinarello C.A., Mantovani A. (2013). The interleukin-1 family: Back to the future. Immunity.

[8] Sims J.E., Smith D.E. (2010). The IL-1 family: Regulators of immunity. Nat. Rev. Immunol..

[9] Dinarello C.A. (2018). Overview of the IL-1 family in innate inflammation and acquired immunity. Immunol. Rev..

[10] Duque G.A., Descoteaux A. (2014). Macrophage cytokines: Involvement in immunity and infectious diseases. Front. Immunol..

[11] Ben-Sasson S.Z., Hu-Li J., Quiel J., Cauchetaux S., Ratner M., Shapira I., Dinarello C.A., Paul W.E. (2009). IL-1 acts directly on CD4 T cells to enhance their antigen-driven expansion and differentiation. Proc. Natl. Acad. Sci. USA.

[12] Wang S., Zhang J., Zhang Y., Yang J., Wang L., Qi Y., Han X., Zhou X., Miao F., Chen T. (2021). Cytokine storm in domestic pigs induced by infection of virulent African swine fever virus. Front. Vet. Sci..

[13] Salguero F.J., Ruiz-Villamor E., Bautista M.J., Sánchez-Cordón P.J., Carrasco L., Gómez-Villamandos J.C. (2002). Changes in macrophages in spleen and lymph nodes during acute African swine fever: Expression of cytokines. Vet. Immunol. Immunopathol..

[14] Salguero F.J., Sánchez-Cordón P.J., Núñez A., Fernández de Marco M., Gómez-Villamandos J.C. (2005). Proinflammatory cytokines induce lymphocyte apoptosis in acute African swine fever infection. J. Comp. Pathol..

[15] Sánchez-Cordón P.J., Jabbar T., Berrezaie M., Chapman D., Reis A., Sastre P., Rueda P., Goatley L., Dixon L.K. (2018). Evaluation of protection induced by immunisation of domestic pigs with deletion mutant African swine fever virus BeninΔMGF by different doses and routes. Vaccine.

[16] Zakaryan H., Cholakyans V., Simonyan L., Misakyan A., Karalova E., Chavushyan A., Karalyan Z. (2015). A study of lymphoid organs and serum proinflammatory cytokines in pigs infected with African swine fever virus genotype II. Arch. Virol..

[17] Radulovic E., Mehinagic K., Wüthrich T., Hilty M., Posthaus H., Summerfield A., Ruggli N., Benarafa C. (2022). The baseline immunological and hygienic status of pigs impact disease severity of African swine fever. PLoS Pathog..

[18] Fan Y., Chen W., Jiang C., Zhang X., Sun Y., Liu R., Wang J., Yang D., Zhao D., Bu Z. (2022). Host Responses to Live-Attenuated ASFV (HLJ/18–7GD). Viruses.

[19] Post J., Weesendorp E., Montoya M., Loeffen W.L. (2017). Influence of age and dose of African swine fever virus infections on clinical outcome and blood parameters in pigs. Viral Immunol..

[20] Reis A.L., Abrams C.C., Goatley L.C., Netherton C., Chapman D.G., Sánchez-Cordón P.J., Dixon L.K. (2016). Deletion of African swine fever virus interferon inhibitors from the genome of a virulent isolate reduces virulence in domestic pigs and induces a protective response. Vaccine.

[21] Carlson J., O’Donnell V., Alfano M., Velazquez Salinas L., Holinka L.G., Krug P.W., Gladue D.P., Higgs S., Borca M.V. (2016). Association of the host immune response with protection using a live attenuated African swine fever virus model. Viruses.

[22] O’Donnell V., Risatti G.R., Holinka L.G., Krug P.W., Carlson J., Velazquez-Salinas L., Azzinaro P.A., Gladue D.P., Borca M.V. (2016). Simultaneous deletion of the 9GL and UK genes from the African swine fever virus Georgia 2007 isolate offers increased safety and protection against homologous challenge. J. Virol..

[23] Li J., Song J., Kang L., Huang L., Zhou S., Hu L., Zheng J., Li C., Zhang X., He X. (2021). pMGF505-7R determines pathogenicity of African swine fever virus infection by inhibiting IL-1β and type I IFN production. PLoS Pathog..

[24] Zhang J., Zhang Y., Chen T., Yang J., Yue H., Wang L., Zhou X., Qi Y., Han X., Ke J. (2021). Deletion of the l7l–l11l genes attenuates ASFV and induces protection against homologous challenge. Viruses.

[25] Gomez-Villamandos J.C., Carrasco L., Bautista M.J., Sierra M.A., Pandalai S.G. (1999). Pathogenesis of African swine fever. The role of monokines. Recent Research Developments in Virology.

[26] Gómez-Villamandos J.C., Bautista M.J., Sánchez-Cordón P.J., Carrasco L. (2013). Pathology of African swine fever: The role of monocyte-macrophage. Virus Res..

[27] Nakanishi K., Yoshimoto T., Tsutsui H., Okamura H. (2001). Interleukin-18 is a unique cytokine that stimulates both Th1 and Th2 responses depending on its cytokine milieu. Cytokine Growth Factor Rev..

[28] Gatti S., Beck J., Fantuzzi G., Bartfai T., Dinarello A.C. (2002). Effect of interleukin-18 on mouse core body temperature. Am. J. Physiol. Regul. Integr. Comp. Physiol..

[29] Keyel P.A. (2014). How is inflammation initiated? Individual influences of IL-1, IL-18 and HMGB1. Cytokine.

[30] Scheller J., Chalaris A., Schmidt-Arras D., Rose-John S. (2011). The pro- and anti-inflammatory properties of the cytokine interleukin-6. Biochim. Biophys. Acta.

[31] Riches D.W., Chan E.D., Winston B.W. (1996). TNF-alpha-induced regulation and signalling in macrophages. Immunobiology.

[32] Vieira S.M., Lemos H.P., Grespan R., Napimoga M.H., Dal-Secco D., Freitas A., Cunha T.M., Verri W.A., Souza Junior D.A., Jamur M.C. (2009). A crucial role for TNF-alpha in mediating neutrophil influx induced by endogenously generated or exogenous chemokines, KC/CXCL1 and LIX/CXCL5. Br. J. Pharmacol..

[33] Griffin G.K., Newton G., Tarrio M.L., Bu D.X., Maganto-Garcia E., Azcutia V., Alcaide P., Grabie N., Luscinskas F.W., Croceet K.J. (2012). IL-17 and TNF-α sustain neutrophil recruitment during inflammation through synergistic effects on endothelial activation. J. Immunol..

[34] Gómez del Moral M., Ortuño E., Fernández-Zapatero P., Alonso F., Alonso C., Ezquerra A., Domínguez J. (1999). African swine fever virus infection induces tumor necrosis factor alpha production: Implications in pathogenesis. J. Virol..

[35] Sánchez-Cordón P.J., Chapman D., Jabbar T., Reis A.L., Goatley L., Netherton C.L., Taylor G., Montoya M., Dixon L. (2017). Different routes and doses influence protection in pigs immunized with the naturally attenuated African swine fever virus isolate OURT88/3. Antivir. Res..

[36] Schäfer A., Franzoni G., Netherton C.L., Hartmann L., Blome S., Blohm U. (2022). Adaptive cellular immunity against African swine fever virus infections. Pathogens.

[37] Morgan D.A., Ruscetti F.W., Gallo R. (1976). Selective in vitro growth of T lymphocytes from normal human bone marrows. Science.

[38] Liao W., Lin J.X., Leonard W.J. (2011). IL-2 family cytokines: New insights into the complex roles of IL-2 as a broad regulator of T helper cell differentiation. Curr. Opin. Immunol..

[39] Vignali D.A., Kuchroo V.K. (2014). IL-12 family cytokines: Immunological playmakers. Nat. Immunol..

[40] Ma X., Trinchieri G. (2001). Regulation of interleukin-12 production in antigen-presenting cells. Adv. Immunol..

[41] Howard M., Farrar J., Hilfiker M., Johnson B., Takatsu K., Hamaoka T., Paul W.E. (1982). Identification of a T cell-derived b cell growth factor distinct from interleukin 2. J. Exp. Med..

[42] Gadani S.P., Cronk C., Norris G.T., Kipnis J. (2012). Interleukin-4: A cytokine to remember. J. Immunol..

[43] Zurawski G., de Vries J.E. (1994). Interleukin 13, an interleukin 4-like cytokine that acts on monocytes and B cells, but not on T cells. Immunol. Today.

[44] Zurawski S.M., Vega F., Huyghe B., Zurawski G. (2018). Receptors for interleukin-13 and interleukin-4 are complex and share a novel component that functions in signal transduction. EMBO J..

[45] Mensikova M., Stepanova H., Faldyna M. (2013). Interleukin-17 in veterinary animal species and its role in various diseases: A review. Cytokine.

[46] Ge Y., Huang M., Yao Y. (2020). Biology of interleukin-17 and its pathophysiological significance in sepsis. Front. Immunol..

[47] Iwakura Y., Ishigame H. (2006). The IL-23/IL-17 axis in inflammation. J. Clin. Investig..

[48] Karalyan Z., Voskanyan H., Ter-Pogossyan Z., Saroyan D., Karalova E. (2016). IL-23/IL-17/G-CSF pathway is associated with granulocyte recruitment to the lung during African swine fever. Vet. Immunol. Immunopathol..

[49] Wu L., Yang B., Yuan X., Hong J., Peng M., Chen J.-L., Song Z. (2021). Regulation and evasion of host immune response by African swine fever virus. Front. Microbiol..

[50] Duvallet E., Semerano L., Assier E., Falgarone G., Boissier M.C. (2011). Interleukin-23: A key cytokine in inflammatory diseases. Ann. Med..

[51] Mosser D.M., Zhang X. (2008). Interleukin-10: New perspectives on an old cytokine. Immunol. Rev..

[52] Saraiva M., O’Garra A. (2010). The regulation of IL-10 production by immune cells. Nat. Rev. Immunol..

[53] Carta T., Razzuoli E., Fruscione F., Zinellu S., Meloni D., Anfossi A., Chessa B., Dei Giudici S., Graham S.P., Oggiano A. (2021). Comparative Phenotypic and Functional Analyses of the Effects of IL-10 or TGF-β on Porcine Macrophages. Animals.

[54] Reis A.L., Goatley L.C., Jabbar T., Sanchez-Cordon P.J., Netherton C.L., Chapman D.A.G., Dixon L.K. (2017). Deletion of the African swine fever virus gene DP148R does not reduce virus replication in culture but reduces virus virulence in pigs and induces high levels of protection against challenge. J. Virol..

[55] Reis A.L., Goatley L.C., Jabbar T., Lopez E., Rathakrishnan A., Dixon L.K. (2020). Deletion of the Gene for the Type I Interferon inhibitor I329L from the attenuated African swine fever virus OURT88/3 strain reduces protection induced in pigs. Vaccines.

[56] Barroso-Arévalo S., Barasona J.A., Cadenas-Fernández E., Sánchez-Vizcaíno J.M. (2021). The Role of interleukine-10 and interferon gamma as potential markers of the evolution of African swine fever virus infection in wild boar. Pathogens.

[57] Schäfer A., Hühr J., Schwaiger T., Dorhoi A., Mettenleiter T.C., Blome S., Schroder C., Blohm U. (2019). Porcine invariant natural killer T cells: Functional profiling and dynamics in steady state and viral infections. Front. Immunol..

[58] Hühr J., Schäfer A., Schwaiger T., Zani L., Sehl J., Mettenleiter T.C., Blome S., Blohm U. (2020). Impaired T-cell responses in domestic pigs and wild boar upon infection with a highly virulent African swine fever virus strain. Transbound. Emerg. Dis..

[59] Sánchez-Cordón P.J., Jabbar T., Chapman D., Dixon L.K., Montoya M. (2020). absence of long-term protection in domestic pigs immunized with attenuated African swine fever virus isolate OURT88/3 or Benin∆MGF correlates with increased levels of regulatory T cells and interleukin-10. J. Virol..

[60] Letterio J.J., Roberts A.B. (1998). Regulation of immune responses by TGF-Β. Annu. Rev. Immunol..

[61] Li M.O., Flavell R.A. (2008). TGF-beta: A master of all T cell trades. Cell.

[62] Morishima N., Mizoguchi I., Takeda K., Mizuguchi J., Yoshimoto T. (2009). TGF-beta is necessary for induction of IL-23R and Th17. Biochem. Biophys. Res. Commun..

[63] Zhang F., Wang H., Wang X., Jiang G., Liu H., Zhang G., Wang H., Fang R., Bu X., Cai S. (2016). TGF-β induces M2-like macrophage polarization via SNAIL-mediated suppression of a pro-inflammatory phenotype. Oncotarget.

[64] Isaacs A., Lindenmann J. (1957). Virus Interference. I. The Interferon. Proc. R. Soc. Lond. B. Biol. Sci..

[65] Samuel C.E. (2001). Antiviral actions of Interferons. Clin. Microbiol. Rev..

[66] Razzuoli E., Armando F., De Paolis L., Ciurkiewicz M., Amadori M. (2022). The swine IFN system in viral infections: Major advances and translational prospects. Pathogens.

[67] Hubel P., Urban C., Bergant V., Schneider W.M., Knauer B., Stukalov A., Scaturro P., Mann A., Brunotte L., Hoffmann H.H. (2019). A protein-interaction network of interferon-stimulated genes extends the innate immune system landscape. Nat. Immunol..

[68] Schneider W.M., Chevillotte M.D., Rice C.M. (2014). Interferon-stimulated genes: A complex web of host defenses. Annu. Rev. Immunol..

[69] Summerfield A. (2012). Viewpoint: Factors involved in type I interferon responses during porcine virus infections. Vet. Immunol. Immunopathol..

[70] Golding J.P., Goatley L., Goodbourn S., Dixon L.K., Taylor G., Netherton C.L. (2016). Sensitivity of African swine fever virus to type I interferon is linked to genes within multigene families 360 and 505. Virology.

[71] Franzoni G., Razzuoli E., Dei Giudici S., Carta T., Galleri G., Zinellu S., Ledda M., Angioi P., Modesto P., Graham S.P. (2020). Comparison of Macrophage Responses to African Swine Fever Viruses Reveals that the NH/P68 Strain is Associated with Enhanced Sensitivity to Type I IFN and Cytokine Responses from Classically Activated Macrophages. Pathogens.

[72] Karalyan Z., Zakaryan H., Sargsyan K., Voskanyan H., Arzumanyan H., Avagyan H., Karalova E. (2012). Interferon status and white blood cells during infection with African swine fever virus in vivo. Vet. Immunol. Immunopathol..

[73] Tau G., Rothman P. (1999). Biologic functions of the IFN-γ receptors. Allergy.

[74] Bhat M.Y., Solanki H.S., Advani J., Khan A.A., Keshava Prasad T.S., Gowda H., Thiyagarajan S. (2018). Comprehensive network map of interferon gamma signaling. J. Cell Commun. Signal.

[75] Konjević G.M., Vuletić A.M., Mirjačić Martinović K.M., Larsen A.K., Jurišić V.B. (2019). The role of cytokines in the regulation of NK cells in the tumor environment. Cytokine.

[76] Portugal R. (2022). ELISpot Assay for the Detection of ASFV-Specific Interferon-Gama (IFN-γ)-Producing Cells. Methods Mol. Biol..

[77] Revilla Y., Pena L., Viñuela E. (1992). Interferon-gamma production by African swine fever virus-specific lymphocytes. Scand J. Immunol..

[78] King K., Chapman D., Argilaguet J.M., Fishbourne E., Hutet E., Cariolet R., Hutchings G., Oura C.A., Netherton C.L., Moffat K. (2011). Protection of European domestic pigs from virulent African isolates of African swine fever virus by experimental immunisation. Vaccine.

[79] Bosch-Camós L., Alonso U., Esteve-Codina A., Chang C.-Y., Martín-Mur B., Accensi F., Muñoz M., Navas M.J., Dabad M., Vidal E. (2022). Cross-protection against African swine fever virus upon intranasal vaccination is associated with an adaptive-innate immune crosstalk. PLoS Pathog..

[80] Argilaguet J.M., Pérez-Martín E., Gallardo C., Salguero F.J., Borrego B., Lacasta A., Accensi F., Díaz I., Nofrarías M., Pujols J. (2011). Enhancing DNA immunization by targeting ASFV antigens to SLA-II bearing cells. Vaccine.

[81] Netherton C.L., Goatley L.C., Reis A.L., Portugal R., Nash R.H., Morgan S.B., Gault L., Nieto R., Norlin V., Gallardo C. (2019). Identification and immunogenicity of African swine fever virus antigens. Front. Immunol..

[82] Goatley L.C., Nash R.H., Andrews C., Hargreaves Z., Tng P., Reis A.L., Graham S.P., Netherton C.L. (2022). Cellular and humoral immune responses after immunisation with low virulent African swine fever virus in the large white inbred Babraham line and outbred domestic pigs. Viruses.

[83] Monteagudo P.L., Lacasta A., López E., Bosch L., Collado J., Pina-Pedrero S., Correa-Fiz F., Accensi F., Navas M.J., Vidal E. (2017). BA71ΔCD2: A new recombinant live attenuated African swine fever virus with cross-protective capabilities. J. Virol..

[84] Goatley L.C., Reis A.L., Portugal R., Goldswain H., Shimmon G.L., Hargreaves Z., Ho C.S., Montoya M., Sánchez-Cordón P.J., Taylor G. (2020). A pool of eight virally vectored African swine fever antigens protect pigs against fatal disease. Vaccines.

[85] Ravilov R.K., Rizvanov A.A., Mingaleev D.N., Galeeva A.G., Zakirova E.Y., Shuralev E.A., Rutland C.S., Khammadov N.I., Efimova M.A. (2022). Viral vector vaccines against ASF: Problems and prospectives. Front. Vet. Sci..

[86] Argilaguet J.M., Pérez-Martín E., Nofrarías M., Gallardo C., Accensi F., Lacasta A., Mora M., Ballester M., Galindo-Cardiel I., López-Soria S. (2012). DNA vaccination partially protects against African swine fever virus lethal challenge in the absence of antibodies. PLoS ONE.

[87] Lacasta A., Ballester M., Monteagudo P.L., Rodriguez J.M., Salas M.L., Accensi F., Pina-Pedrero S., Bensaid A., Argilaguet J., Lopez-Soria S. (2014). Expression library immunization can confer protection against lethal challenge with African swine fever virus. J. Virol..

[88] Lacasta A., Monteagudo P.L., Jiménez-Marín Á., Accensi F., Ballester M., Argilaguet J., Galindo-Cardiel I., Segalés J., Salas M.L., Domínguez J. (2015). Live attenuated African swine fever viruses as ideal tools to dissect the mechanisms involved in viral pathogenesis and immune protection. Vet. Res..

[89] Takamatsu H.H., Denyer M.S., Lacasta A., Stirling C.M., Argilaguet J.M., Netherton C.L., Oura C.A., Martins C., Rodríguez F. (2013). Cellular immunity in ASFV responses. Virus Res..

[90] Comerford I., McColl S.R. (2011). Mini-review series: Focus on chemokines. Immunol. Cell Biol..

[91] Alcami A., Lira S.A. (2010). Modulation of chemokine activity by viruses. Curr. Opin. Immunol..

[92] Beste M.T., Lomakina E.B., Hammer D.A., Waugh R.E. (2015). Immobilized IL-8 Triggers phagocytosis and dynamic changes in membrane microtopology in human neutrophils. Ann. Biomed. Eng..

[93] Hedges J.C., Singer C.A., Gerthoffer W.T. (2000). Mitogen-activated protein kinases regulate cytokine gene expression in human airway myocytes. Am. J. Respir. Cell. Mol. Biol..

[94] Fishbourne E., Hutet E., Abrams C., Cariolet R., Le Potier M.F., Takamatsu H.H., Dixon L.K. (2013). Increase in chemokines CXCL10 and CCL2 in blood from pigs infected with high compared to low virulence African swine fever virus isolates. Vet. Res..

[95] Antonelli A., Ferrari S.M., Giuggioli D., Ferrannini E., Ferri C., Fallahi P. (2014). Chemokine (C-X-C motif) ligand (CXCL)10 in autoimmune diseases. Autoimmun. Rev..

[96] Laing K.J., Secombes C.J. (2004). Chemokines. Dev. Comp. Immunol..

[97] Dufour J.H., Dziejman M., Liu M.T., Leung J.H., Lane T.E., Luster A.D. (2002). IFN-gamma-inducible protein 10 (IP-10; CXCL10)-deficient mice reveal a role for IP-10 in effector T cell generation and trafficking. J. Immunol..

[98] Bleul C.C., Fuhlbrigge R.C., Casasnovas J.M., Aiuti A., Springer T.A. (1996). A highly efficacious lymphocyte chemoattractant, stromal cell-derived factor 1 (SDF-1). J. Exp. Med..

[99] Zheng H., Fu G., Dai T., Huang H. (2007). Migration of endothelial progenitor cells mediated by stromal cell-derived factor-1alpha/CXCR4 via PI3K/Akt/eNOS signal transduction pathway. J. Cardiovasc. Pharmacol..

[100] Guo F., Wang Y., Liu J., Mok S.C., Xue F., Zhang W. (2016). CXCL12/CXCR4: A symbiotic bridge linking cancer cells and their stromal neighbours in oncogenic communication networks. Oncogene.

[101] Tatoyan M.R., Ter-Pogossyan Z.R., Semerjyan A.B., Gevorgyan V.S., Karalyan N.Y., Sahakyan C.T., Mkrtchyan G.L., Gazaryan H.K., Avagyan H.R., Karalyan Z.A. (2019). Serum concentrations of vascular endothelial growth factor, stromal cell-derived factor, nitric oxide and endothelial dna proliferation in development of microvascular pathology in acute African swine fever. J. Comp. Pathol..

[102] Deshmane S.L., Kremlev S., Amini S., Sawaya B.E. (2009). Monocyte chemoattractant protein-1 (MCP-1): An overview. J. Interferon Cytokine Res..

[103] Xu L.L., Warren M.K., Rose W.L., Gong W., Wang J.M. (1996). Human recombinant monocyte chemotactic protein and other c-c chemokines bind and induce directional migration of dendritic cells in vitro. J. Leukoc. Biol..

[104] Carr M.W., Roth S.J., Luther E., Rose S.S., Springer T.A. (1994). Monocyte chemoattractant protein 1 acts as a T-lymphocyte chemoattractant. Proc. Natl. Acad. Sci. USA.

[105] Sherry B., Tekamp-Olson P., Gallegos C., Bauer D., Davatelis G., Wolpe S.D., Masiarz F., Coit D., Ceramiet A. (1988). Resolution of the two components of macrophage inflammatory protein 1, and cloning and characterization of one of those components, macrophage inflammatory protein 1beta. J. Exp. Med..

[106] Wolpe S.D., Davatelis G., Sherry B., Beutler B., Hesse D.G., Nguyen H.T., Moldawer L.L., Nathan C.F., Lowry S.F., Cerami A. (1988). Macrophages secrete a novel heparin-binding protein with inflammatory and neutrophil chemokinetic properties. J. Exp. Med..

[107] Menten P., Wuyts A., Van Damme J. (2002). Macrophage inflammatory protein-1. Cytokine Growth Factor Rev..

[108] Zeng Z., Lan T., Wei Y., Wei X. (2022). CCL5/CCR5 axis in human diseases and related treatments. Genes Dis..

[109] Maghazachi A.A., Al-Aoukaty A., Schall T.J. (1996). CC chemokines induce the generation of killer cells from CD56+ cells. Eur. J. Immunol..

[110] Demetri G.D., Griffin J.D. (1991). Granulocyte colony stimulating factor and its receptor. Blood.

[111] Kato T., Ando H., Ukena K., Nagata S. (2021). Subchapter 40C—Granulocyte colony-stimulating factor. Handbook of Hormones. Comparative Endocrinology for Basic and Clinical Research.

[112] van de Geijn G.J., Aarts L.H., Erkeland S.J., Prasher J.M., Touw I.P. (2003). Granulocyte colony-stimulating factor and its receptor in normal hematopoietic cell development and myeloid disease. Rev. Physiol. Biochem. Pharmacol..

[113] Zhan Y., Lew A.M., Chopin M. (2019). The pleiotropic effects of the Gm-Csf rheostat on myeloid cell differentiation and function: More than a numbers game. Front. Immunol..

[114] Fossati G., Mazzucchelli I., Gritti D., Ricevuti G., Edwards S., Moulding D., Rossi M.L. (1998). In vitro effects of GM-CSF on mature peripheral blood neutrophils. Int. J. Mol. Med..

[115] Gasson J.C. (1991). Molecular physiology of granulocyte-macrophage colony-stimulating factor. Blood.

[116] Potter H., Boyd T.D., Clarke P., Pelak V.S., Tyler K.L. (2020). Recruiting the innate immune system with GM-CSF to fight viral diseases, including West Nile Virus encephalitis and COVID-19. F1000Research.

[117] Chung A.S., Ferrara N. (2011). Developmental and pathological angiogenesis. Annu. Rev. Cell. Dev. Biol..

[118] Maniscalco W.M., D’Angio C.T., Laurent G.J., Shapiro S.D. (2006). Vascular endothelial growth factor. Encyclopedia of Respiratory Medicine.

[119] Carrasco L., Chacón-M de Lara F., Martín de las Mulas J., Gómez-Villamandos J.C., Sierra M.A., Villeda C.J., Wilkinson P.J. (1997). Ultrastructural changes related to the lymph node haemorrhages in acute African swine fever. Res. Vet. Sci..

[120] Gómez-Villamandos J.C., Hervás J., Méndez A., Carrasco L., Martín de las Mulas J., Villeda C.J., Wilkinson P.J., Sierra M.A. (1995). Experimental African swine fever: Apoptosis of lymphocytes and virus replication in other cells. J. Gen. Virol..

[121] Carrasco L., de Lara F.C., Martín de las Mulas J., Gómez-Villamandos J.C., Pérez J., Wilkinson P.J., Sierra M.A. (1996). Apoptosis in lymph nodes in acute African swine fever. J. Comp. Pathol..

[122] Ramiro-Ibáñez F., Ortega A., Brun A., Escribano J.M., Alonso C. (1996). Apoptosis: A mechanism of cell killing and lymphoid organ impairment during acute African swine fever virus infection. J. Gen. Virol..

[123] Oura C.A., Powell P.P., Parkhouse R.M. (1998). African swine fever: A disease characterized by apoptosis. J. Gen. Virol..

[124] Nagata S. (1997). Apoptosis by death factor. Cell.

[125] Hensley L.E., Young H.A., Jahrling P.B., Geisbert T.W. (2002). Proinflammatory response during Ebola virus infection of primate models: Possible involvement of the tumor necrosis factor receptor superfamily. Immunol. Lett..

[126] Cardier J.E., Marino E., Romano E., Taylor P., Liprandi F., Bosch N., Rothman A.L. (2005). Proinflammatory factors present in sera from patients with acute dengue infection induce activation and apoptosis of human microvascular endothelial cells: Possible role of TNF-alpha in endothelial cell damage in dengue. Cytokine.

[127] Nicholas S.A., Oniku A.E., Sumbayev V.V. (2010). Myeloid cell death associated with Toll-like receptor 7/8-mediated inflammatory response. Implication of ASK1, HIF-1 alpha, IL-1 beta and TNF-alpha. Mol. Immunol..

[128] Castillo J.A., Urcuqui-Inchima S. (2018). Mechanisms of monocyte cell death triggered by dengue virus infection. Apoptosis.

[129] Neilan J.G., Lu Z., Afonso C.L., Kutish G.F., Sussman M.D., Rock D.L. (1993). An African swine fever virus gene with similarity to the proto-oncogene bcl-2 and the Epstein-Barr virus gene BHRF1. J. Virol..

[130] Yáñez R.J., Rodríguez J.M., Nogal M.L., Yuste L., Enríquez C., Rodriguez J.F., Viñuela E. (1995). Analysis of the complete nucleotide sequence of African swine fever virus. Virology.

[131] Afonso C.L., Neilan J.G., Kutish G.F., Rock D.L. (1996). An African swine fever virus Bc1-2 homolog, 5-HL, suppresses apoptotic cell death. J. Virol..

[132] Casal I., Enjuanes L., Viñuela E. (1984). Porcine leukocyte cellular subsets sensitive to African swine fever virus in vitro. J. Virol..

[133] Salguero F.J., Sánchez-Cordón P.J., Sierra M.A., Jover A., Núñez A., Gómez-Villamandos J.C. (2004). Apoptosis of thymocytes in experimental African Swine Fever virus infection. Histol. Histopathol..

[134] Fernández de Marco M., Salguero F.J., Bautista M.J., Núñez A., Sánchez-Cordón P.J., Gómez-Villamandos J.C. (2007). An immunohistochemical study of the tonsils in pigs with acute African swine fever virus infection. Res. Vet. Sci..

[135] Sánchez-Cordón P.J., Romero-Trevejo J.L., Pedrera M., Sánchez-Vizcaíno J.M., Bautista M.J., Gómez-Villamandos J.C. (2008). Role of hepatic macrophages during the viral haemorrhagic fever induced by African Swine Fever Virus. Histol. Histopathol..

[136] Shimizu H., Mitomo K., Watanabe T., Okamoto S., Yamamoto K. (1990). Involvement of a NF-kappa B-like transcription factor in the activation of the interleukin-6 gene by inflammatory lymphokines. Mol. Cell Biol..

[137] Ginsberg H.S., Moldawer L.L., Sehgal P.B., Redington M., Kilian P.L., Chanock R.M., Prince G.A. (1991). A mouse model for investigating the molecular pathogenesis of adenovirus pneumonia. Proc. Natl. Acad. Sci. USA.

[138] Sánchez-Cordón P.J., Cerón J.J., Núñez A., Martínez-Subiela S., Pedrera M., Romero-Trevejo J.L., Garrido M.R., Gómez-Villamandos J.C. (2007). Serum concentrations of C-reactive protein, serum amyloid A and haptoglobin in pigs inoculated with African swine fever or classical swine fever viruses. Am. J. Vet. Res..

[139] Carrasco L., de Lara F.C., Gómez-Villamandos J.C., Bautista M.J., Villeda C.J., Wilkinson P.J., Sierra M.A. (1996). The pathogenic role of pulmonary intravascular macrophages in acute African swine fever. Res. Vet. Sci..

[140] Carrasco L., Núñez A., Salguero F.J., Díaz San Segundo F., Sánchez-Cordón P., Gómez-Villamandos J.C., Sierra M.A. (2002). African swine fever: Expression of interleukin-1 alpha and tumour necrosis factor-alpha by pulmonary intravascular macrophages. J. Comp. Pathol..

[141] Gómez-Villamandos J.C., Hervás J., Méndez A., Carrasco L., Villeda C.J., Wilkinson P.J., Sierra M.A. (1995). Pathological changes in the renal interstitial capillaries of pigs inoculated with two different strains of African swine fever virus. J. Comp. Pathol..

[142] Mekonnen T., Salguero F.J., Ruiz-Villamor L., Carrasco E., Gómez-Villamandos J.C. IL-6 expression in liver and kidney of pigs inoculated with African swine fever virus (isolate E-70). Proceedings of the 12th Meeting of the Spanish Society of Veterinary Pathology.

[143] Mekonnen T. (2001). Expression of Monokines in the Liver and Kidney of Pigs Inoculated with African Swine Fever Virus. Ph.D. Thesis.

[144] Sprague A.H., Khalil R.A. (2009). Inflammatory cytokines in vascular dysfunction and vascular disease. Biochem. Pharmacol..

[145] Fan W., Cao Y., Jiao P., Yu P., Zhang H., Chen T., Zhou X., Qi Y., Sun L., Liu D. (2022). Synergistic effect of the responses of different tissues against African swine fever virus. Transbound. Emerg. Dis..

[146] Machuka E.M., Juma J., Muigai A.W.T., Amimo J.O., Pelle R., Abworo E.O. (2022). Transcriptome profile of spleen tissues from locally-adapted Kenyan pigs (Sus scrofa) experimentally infected with three varying doses of a highly virulent African swine fever virus genotype IX isolate: Ken12/busia.1 (ken-1033). BMC Genom..

[147] Ding M., Dang W., Liu H., Xu F., Huang H., Sunkang Y., Li T., Pei J., Liu X., Zhang Y. (2022). Combinational deletions of MGF360-9L and MGF505-7R Attenuated Highly Virulent African swine fever virus and conferred protection against homologous challenge. J. Virol..

[148] Karki R., Kanneganti T.D. (2021). The ‘cytokine storm’: Molecular mechanisms and therapeutic prospects. Trends Immunol..

[149] Paessler S., Walker D.H. (2013). Pathogenesis of the viral hemorrhagic fevers. Annu. Rev. Pathol..

